# Mapping the FF domain folding pathway via structures of transiently populated folding intermediates

**DOI:** 10.1073/pnas.2416682121

**Published:** 2024-12-04

**Authors:** Debajyoti De, Nemika Thapliyal, Ved Prakash Tiwari, Yuki Toyama, D. Flemming Hansen, Lewis E. Kay, Pramodh Vallurupalli

**Affiliations:** ^a^Tata Institute of Fundamental Research Hyderabad, Ranga Reddy District, Hyderabad 500046, India; ^b^Department of Molecular Genetics, University of Toronto, Toronto M5S 1A8, Canada; ^c^Department of Chemistry, University of Toronto, Toronto, ON M5S 3H6, Canada; ^d^Department of Biochemistry, University of Toronto, Toronto, ON M5S 1A8, Canada; ^e^Center for Biosystems Dynamics Research, RIKEN, Kanagawa 230-0045, Japan; ^f^Department of Structural and Molecular Biology, Division of Biosciences, University College London, London WC1E 6BT, United Kingdom; ^g^The Francis Crick Institute, London NW1 1AT, United Kingdom

**Keywords:** protein folding, sparse folding intermediate structures, urea *m*-values, chemical exchange saturation transfer (CEST), NMR

## Abstract

Despite the tremendous accomplishments of AlpaFold2/3 in predicting biomolecular structure, the protein folding problem remains unsolved in the sense that accurate atomistic models of how protein molecules fold into their native conformations from an unfolded ensemble are still elusive. Here, using chemical exchange saturation transfer (CEST) NMR experiments and a comprehensive four-state kinetic model of the folding trajectory of a 71 residue four-helix bundle FF domain from human HYPA/FBP11 we present an atomic resolution structure of a transiently formed intermediate, I2, that along with the structure of a second intermediate, I1, provides a description of the FF domain folding trajectory. By recording CEST profiles as a function of urea concentration the extent of compaction along the folding pathway is evaluated. Our data establish that unlike the partially disordered I1 state, the I2 intermediate that is also formed before the rate-limiting folding barrier is well ordered and compact like the native conformer, while retaining nonnative interactions similar to those found in I1. The slow-interconversion from I2 to F, involving changes in secondary structure and the breaking of nonnative interactions, proceeds via a compact transition-state. Interestingly, the native state of the FF1 domain from human p190-A Rho GAP resembles the I2 conformation, suggesting that well-ordered folding intermediates can be repurposed by nature in structurally related proteins to assume functional roles. It is anticipated that the strategy for elucidation of sparsely populated and transiently formed structures of intermediates along kinetic pathways described here will be of use in other studies of protein dynamics.

## Significance

Many protein molecules fold into well-defined functional structures. Although machine learning approaches enable accurate prediction of many of these structures, the mechanisms by which they fold remain elusive. Using solution NMR spectroscopy, we describe the folding trajectory of a protein domain at atomic resolution. We show that the rapid collapse from the unfolded ensemble results in a folding intermediate with some elements of native structure, but also with nonnative contacts. Many of these are retained in a more mature and highly compact second intermediate which transitions to the native conformer without expansion of the polypeptide chain. The strategy for structure elucidation of sparse intermediates described here is likely to find application in studies of other dynamic systems.

It is now well established that even small single-domain proteins often fold via intermediates (1–4) and a detailed description of each folding trajectory requires, therefore, the determination of atomic resolution structures of all the folding intermediates along the kinetic pathway. Determining the folding mechanism from experimental data and subsequently generating atomic resolution models of the relevant intermediates remains a challenge (1, 5). As a result, detailed folding pathways are not often available even for small proteins that have served as model systems and that have been extensively studied using multiple techniques. Detection of intermediates and the determination of an appropriate exchange mechanism for a given folding reaction is challenging. First, intermediates are often sparsely populated at equilibrium. While the populations of folded (F) and unfolded (U) states can be manipulated in a controlled manner by varying experimental conditions, such as temperature and denaturant concentration, intermediates frequently remain only marginally populated. Second, it is often difficult to distinguish intermediates from each other, as changes in secondary structure including helix elongation, register shifts in β sheets, and formation of new tertiary contacts can escape detection by optical probes. Thus, both detecting and, subsequently, distinguishing between the various intermediates requires techniques that are sensitive to residue or atomic level changes in structure, such as solution NMR spectroscopy. Although solution NMR experiments can be used to probe the structure and dynamics at almost every site in a protein molecule, signals from sparsely populated states are not visible in traditional NMR spectra (6). Nonetheless, over the last two decades, a number of NMR relaxation experiments have been developed to detect minor states of proteins in exchange with the dominant visible state, with lifetimes of the minor conformers ranging from ~10 µs to ~100 ms and populations that can be as low as ~0.1% in some cases (7–12). Relaxation-based NMR experiments have been used to study biomolecular conformational dynamics associated with processes such as folding, aggregation, and molecular recognition (5, 8, 9, 13–17), and these techniques have been extended (10, 18–20) to determine structures of transiently populated minor states in favorable cases (3, 18, 20–26). A third, and more subtle, difficulty with detecting short-lived, sparse states on the exchange pathway of interest occurs when both slow and fast processes are involved, with rapidly exchanging intermediates on the opposite side of the major transition state from the dominant conformer (1, 27). In its simplest form, an example would be an exchange process $F\underset{k_{\mathrm{FI}}}{\overset{k_{\mathrm{IF}}}{⇋}}I\underset{k_{\mathrm{IU}}}{\overset{k_{\mathrm{UI}}}{⇋}}U$ in which folding proceeds via an intermediate state I, with the populations of all states, $p_{i}$, ordered as $p_{F}\gg p_{U}\gg p_{I}$ and where the global folding transition state separates F from I ($k_{ex,IU}\gg k_{ex,FI}$; $k_{ex,JK}=k_{\mathrm{JK}}+k_{\mathrm{KJ}}$). In this case, the exchange process probed using the dominant F state can appear as two-state, F⇋U, missing the crucial folding intermediate I. The inability to detect early folding intermediates imposes a severe limitation on the description of the folding of even small proteins (1).

Recently, we showed that chemical exchange saturation transfer (CEST) NMR experiments (10, 28–30) can be used to inform on exchange processes in which minor states rapidly interconvert with each other (“minor exchange”), exposing the “blind spot” that has challenged other methodologies, as described above (31, 32) (*SI Appendix* *f*or details of CEST, *SI Appendix*, Fig. S1). In addition to extracting a folding model and exchange rates, importantly, chemical shifts (ϖ) of nuclei in the different states can also be obtained via the CEST approach, potentially allowing the determination of structural models of all intermediates along a protein folding pathway.

**Fig. 1.**
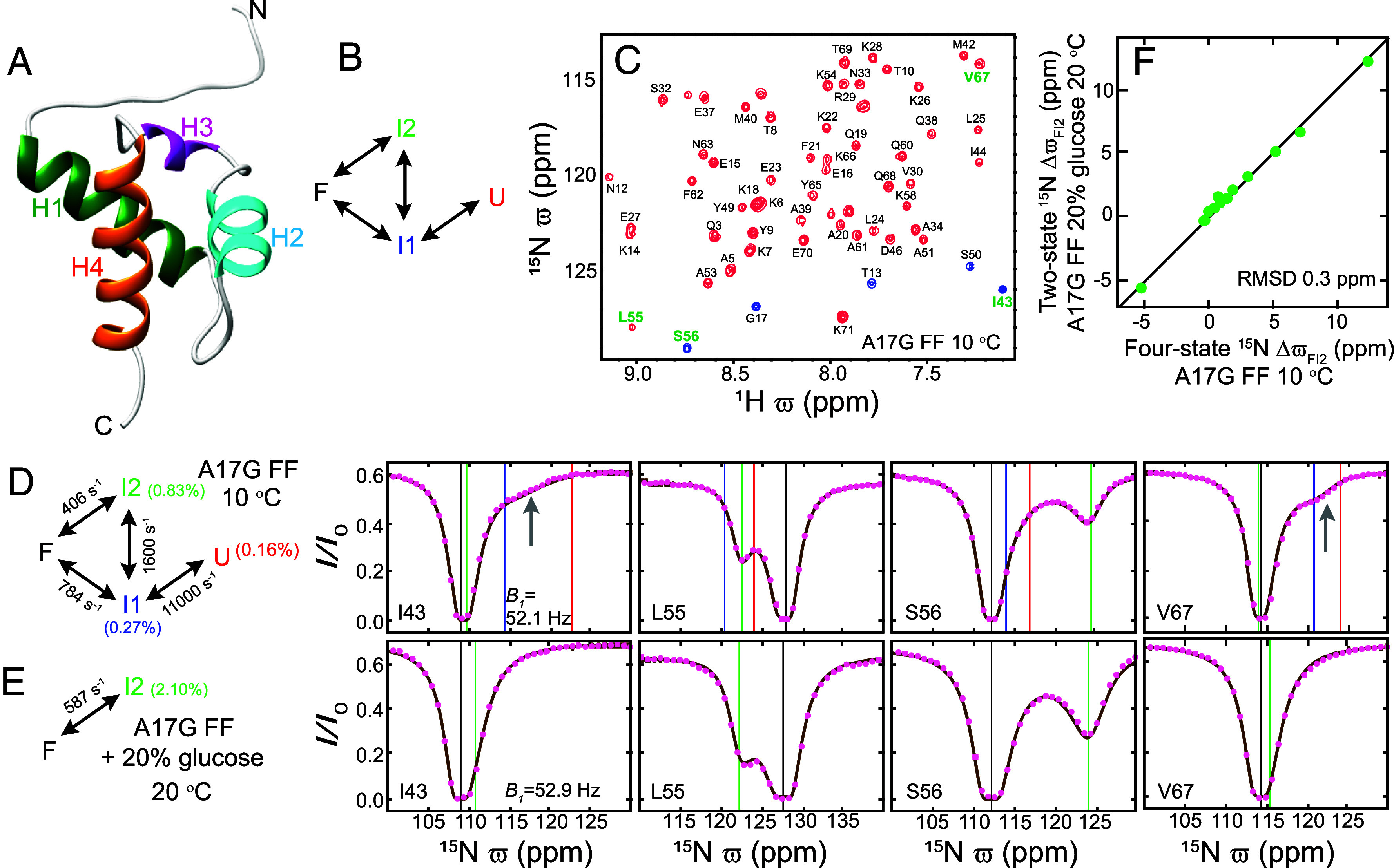
Simplifying FF domain exchange to obtain I2 state chemical shifts. (*A*) The native (F) state structure [PDB: 1UZC] (33) of the WT FF domain contains a 3_10_ helix, H3 (P47–S50: magenta), and three α helices, H1 (K14–E27; dark green), H2 (W36–I43; cyan), and H4 (L55–Q68: orange). Helix boundaries were obtained using TALOS-N (37). (*B*) Schematic illustration of the ^15^N CEST derived FF domain folding model showing that FF folds via two intermediates I1 and I2 along two pathways. (*C*) Amide ^1^H-^15^N correlation map of A17G FF (16.4 T; 10 °C). Peaks aliased in the ^15^N dimension are shown in blue. Peak assignments are indicated. (*D*) Amide ^15^N CEST profiles (*B_1_* = 52.1 Hz, *T_EX_* = 400 ms, 16.4 T, 10 °C) from I43, L55, S56, and V67 in A17G FF. The data were analyzed using the four-state exchange model shown on the left, to produce best-fit exchange parameters (also indicated; *SI Appendix*, Table S4). The vertical black, blue, green, and red lines correspond to the fitted $\varpi_{F}$, $\varpi_{I1}$, $\varpi_{I2}$, and $\varpi_{U}$ values, respectively. The gray arrow points to the dip that arises due to rapid I1 ⇋ U exchange. (*E*) Amide ^15^N CEST profiles (*B_1_* = 52.9 Hz, *T_EX_* = 400 ms, 16.4 T, 20 °C) from I43, L55, S56, and V67 recorded after adding 20% (w/v) glucose to the sample. The data were analyzed using the two-state exchange model shown, generating the best-fit exchange parameters as indicated. The vertical black and green lines correspond to the fitted $\varpi_{F}$ and $\varpi_{I2}$ values, respectively. In (*D*) and (*E*) the experimental data are shown using pink circles while the brown line is calculated using the best-fit parameters. (*F*) The ${\Delta \varpi }_{FI2}$ values obtained by analyzing the amide ^15^N CEST data using a four-state model in the absence of glucose (*D*) and a two-state model in the presence of 20% glucose (*E*) are very similar, showing that accurate $\varpi_{I2}$ values can be obtained from a simple two-state analysis of A17G FF CEST data recorded in the presence of 20% glucose.

Here we illustrate that elucidation of multiple structural intermediates is indeed, possible, using as an example the 71 residue four-helix bundle FF domain from human HYPA/FBP11 (Fig. 1*A*), a model system to study protein folding. In the F state helices H1, H2, H3, and H4 adopt an α-α-3_10_-α topology in which H3 is the 3_10_ helix (33), while the first ten residues (N-terminal tail) are not part of the folded FF domain and are not discussed further. Extensive folding studies of wild-type (WT) and various mutants of the FF domain using stop-flow, temperature-jump, and Carr-Purcell-Meiboom-Gill (CPMG) relaxation dispersion NMR experiments have shown that the protein folds via a sparsely populated intermediate (4, 34–36). Interestingly, unlike for all other FF domain variants, a folding intermediate could not be detected for the A39G FF mutant from stop-flow data (35, 36), but only an exchange process in which the major F state interconverts slowly with the minor U state (*p_U_* ~1% at 1 °C). However, on the basis of ^15^N CEST experiments (*SI Appendix*) in which both the position and linewidths of minor state dips were used to detect other, even more sparsely populated, states in rapid exchange with one another (32) the A39G FF domain was found to fold along two paths via two intermediates, I1 and I2 (Fig. 1*B*). Here, the U state exchanges rapidly with I1, $k_{ex,I1U}$ ~ 8,500 s^−1^, while the exchange between I1 and I2 is somewhat slower with $k_{ex,I1I2}$ ~ 1,600 s^−1^. I1 and I2 then exchange slowly with F ($k_{ex,FI1}$ ~ 150 s^−1^ & $k_{ex,FI2}$ ~ 350 s^−1^). As the CEST derived I1 state chemical shifts ($\varpi_{I1}$) (31, 32) are in excellent agreement with those obtained previously for the folding intermediate of WT FF (3), it can be concluded that both the WT and A39G FF domains fold via a similar I1 intermediate. Further CEST studies, in which the folding of A17G and WT FF were studied using ^15^N CEST, confirmed that they also fold via both I1 and I2, providing strong evidence that all the FF domain variants fold via similar pathways that can be described in terms of the same four states (31, 32).

Herein we provide structural models for the complete FF domain folding pathway. Analysis of ^15^N, ^1^H^N^, ^1^H^α^, ^13^C^α^, ^13^C^O^, ^13^C^β^, and methyl ^1^H and ^13^C CEST profiles allowed us to obtain a large number of chemical shifts for the I2 state which form the basis for determination of the structure of this intermediate using the CS-ROSETTA program (38). The structure of I2 is validated via experiments carried out under conditions where its population is significantly enhanced (~25%). We further recorded CEST experiments as a function of urea concentration, and interpret the resulting urea-based *m*-values for all sparsely populated states and the four transition-states in terms of compaction along the folding trajectory. Together with the structure of the I1 state that has been determined using CPMG experiments recorded on the WT FF domain under conditions in which the major F state interconverts with the minor I1 conformer (3), the presented I2 structure allows a detailed description of FF domain folding at atomic-resolution. Unlike the “less mature” I1 state where helix H4 is disordered and the overall structure is loosely packed, the CEST derived I2 state structure is fully ordered and as compact as the folded conformer, F, while still retaining some of the nonnative features present in the I1 state. The *m*-value of the transition-state between F and I2 shows that the interconversion between these compact fully ordered states, involving changes in secondary structure and breaking of either native (F to I2) or nonnative (I2 to F) contacts, proceeds without expansion of the protein. Our study highlights that, at least in some cases, it is possible to obtain structural descriptions of multiple “invisible” intermediates along a reaction, potentially opening up possibilities for a detailed description of the underlying biological processes involved and providing insights into how one might manipulate these processes in cases where they can be associated with function or, alternatively, misfunction.

## Results

The determination of structural models for “invisible” protein conformers (so-called excited conformational states) requires chemical shift measurements at a large number of backbone sites (38–40). These shifts must be obtained through relaxation dispersion- or CEST-based experiments, as crosspeaks for excited states are not observed in standard ^1^H-^15^N or ^1^H-^13^C experiments. As the FF domain folds via two sparse and transiently formed intermediates along a pair of kinetic pathways (32), the resulting relaxation data must be analyzed in a four-state manner, involving in the most general case twelve different rate constants to reliably obtain chemical shifts of the rare conformers. In the case of the FF domain, it could be established, based on analysis of ^15^N CEST data, that a simplified four-state model was sufficient (Fig. 1*B*) (32), allowing extraction of ^15^N chemical shifts of each conformer (U, I1, and I2). However, it is unlikely that similar four-state fits of less sensitive CEST data (relative to ^15^N) recorded at backbone ^1^H and ^13^C sites and sidechain ^13^C^β^ carbons would be of sufficient quality to allow robust estimates of these additional chemical shifts. An additional complicating factor relative to ^15^N data is that dips in ^13^C CEST profiles recorded using uniformly ^13^C labeled samples are broadened from ^1^J_CC_ couplings (41–43), while dip linewidths in ^1^H CEST profiles are increased due to ^1^H-^1^H scalar and dipolar interactions (44). Hence there is a need to “simplify” the exchange process to robustly obtain the complete set of I2 state chemical shifts necessary for structural studies.

### Simplifying Exchange to Determine Chemical Shifts of the Sparsely Populated Folding Intermediate I2.

Earlier we had noticed that dips derived from the I2 state are visible in ^15^N CEST profiles recorded on A17G FF because this variant populates the I2 state to a greater extent than is the case for A39G FF or WT FF domains (32). The amide ^1^H-^15^N correlation map of A17G FF is well resolved (Fig. 1*C*) and representative amide ^15^N CEST profiles from I43, L55, S56, and V67 are shown in Fig. 1*D*. The ^15^N CEST data (*B_1_* = 26, 52.1, 104.1, and 208.3 Hz) were analyzed using a four-state exchange model (Fig. 1*B*; $\chi_{\mathrm{red}}^{2}$ ~ 0.7, *k_ex,FI1_* = 784 ± 67 s^−1^, *k_ex,FI2_* = 406 ± 5 s^−1^, *k_ex,I1I2_* = 1,600 ± 113 s^−1^, *k_ex,I1U_* = 11,000 ± 1,064 s^−1^, *p_I1_* = 0.27 ± 0.01%, *p_I2_* = 0.83 ± 0.01% and *p_U_* = 0.16 ± 0.02%). In Fig. 1*D* the CEST derived ^15^N $\varpi_{F}$, $\varpi_{I2}$_,_ $\varpi_{I1}$, and $\varpi_{U}$ chemical shift values (ppm) are shown on the CEST profiles using black, green, blue, and red vertical lines, respectively. The shifts agree well with those obtained previously for A39G FF (*SI Appendix*, Fig. S2), once again confirming that the different FF variants fold via the same intermediates. Notably, the ^15^N CEST profiles for I43 and V67 contain a broad dip (indicated by the gray arrows in Fig. 1*D*) between the blue and red lines, arising due to the fast exchange between the I1 and U states. On the other hand, CEST profiles from L55 and S56 contain a clear dip near the green line due to the I2 state. As additives such as glucose and glycerol stabilize folded protein states (F) (45) we added glucose to the buffer in the hopes of reducing the populations of both U and the partially disordered I1 state relative to I2. Indeed, the addition of 20% (w/v) glucose reduces both *p_I1_* and *p_U_* as can be seen from the disappearance of the dips arising from the I1 ⇋ U interconversion (compare CEST profiles from I43 and V67 in Fig. 1 *D* and *E*), while the dips arising from the I2 state remain intact (compare L55 and S56 in Fig. 1 *D* and *E*). The 20% glucose A17G FF ^15^N CEST data is well fit using a two-site exchange model ($\chi_{\mathrm{red}}^{2}$ ~ 1.1; *B_1_* = 26.4 & 52.9 Hz) with $k_{ex,FI2}$ = 587 ± 12 s^−1^ and $p_{I2}$ = 2.1 ± 0.01%, and, importantly, the I2 state chemical shifts obtained from the two-state analysis agree very well with the corresponding values from four-state fits of CEST data recorded in the absence of glucose (RMSD 0.3 ppm; Fig. 1*F*). Thus, the 20% glucose buffer stabilizes the F and I2 states, simplifying the four-state exchange process to one which can be described in terms of F and I2, so that the I2 state chemical shifts can be obtained accurately. Further, in the presence of 20% glucose, the population of I2 increases from ~1% at 10 °C to ~2% at 20 °C, which is why the higher temperature was chosen for the two-state analysis above, as opposed to our initial study in the absence of glucose which was conducted at 10 °C.

### Assembling a Near-Complete Set of I2 State Backbone Chemical Shifts.

**Fig. 2.**
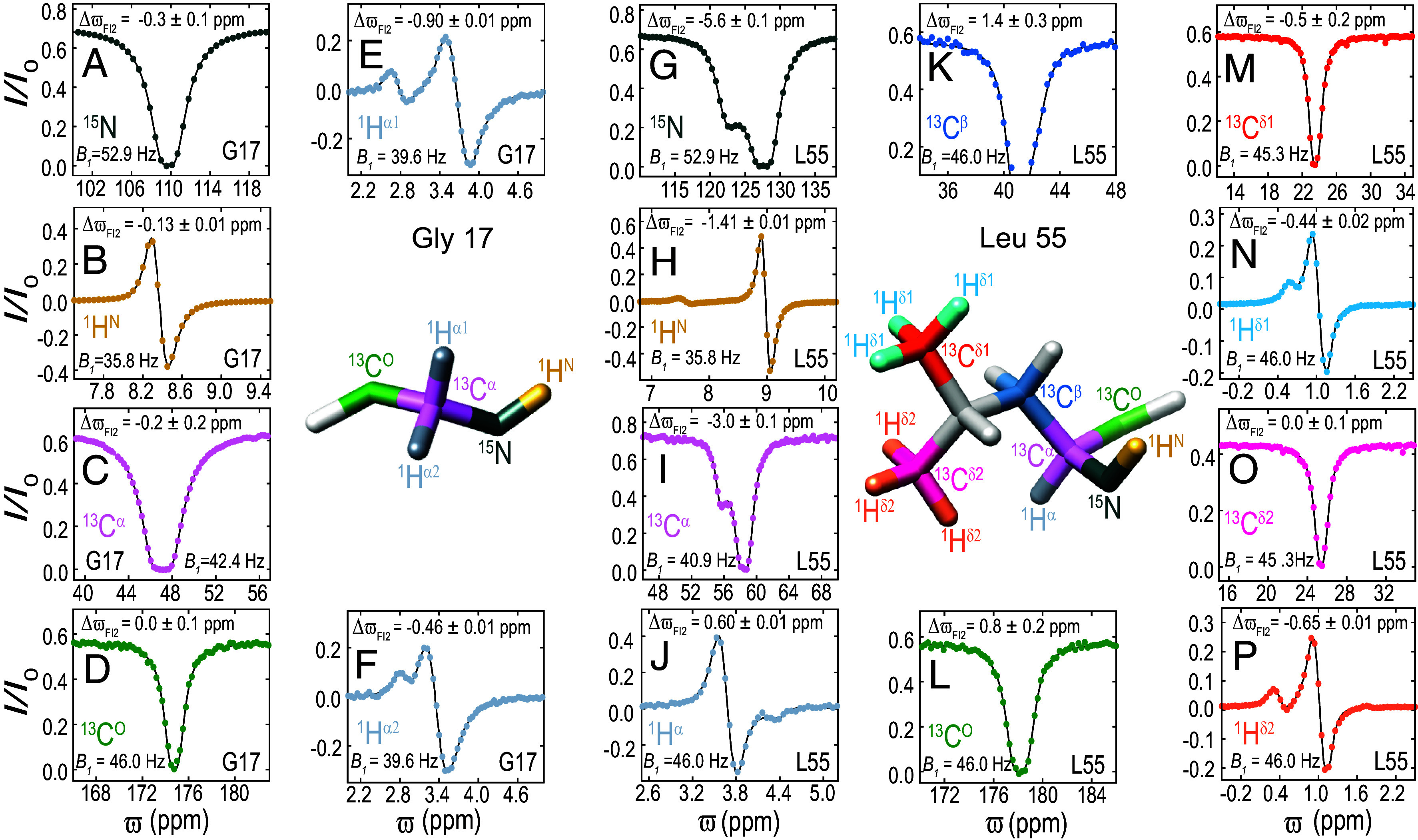
Reconstructing NMR spectra of the sparsely populated I2 state of the FF domain. ^1^H, ^15^N, and ^13^C CEST profiles from various sites in G17 (*A–F*) and L55 (*G–P*) recorded using A17G FF samples in 20% [^2^H] glucose buffer (16.4 T, 20 °C). The *B_1_* value used to record the CEST data and the best-fit ${\Delta \varpi }_{FI2}$ value are indicated for each site. Experimental data are shown using colored circles, while the black line is calculated using best fit two-state parameters. Experimental details are given in *SI Appendix*.

Having established that accurate $\varpi_{I2}$ values can be obtained from a two-state analysis of CEST data measured on A17G FF samples in 20% glucose buffer, we next recorded ^1^H and ^13^C CEST profiles to obtain minor state chemical shifts at nearly all backbone and ^13^C^β^ sites using CEST experiments that have been developed over the last decade (30, 42, 43, 46–48). Analysis of these CEST profiles in a two-state manner resulted in an extensive set of backbone I2 state chemical shifts (Fig. 2 and *SI Appendix*, Tables S1–S3). Amide ^1^H^N^ and ^15^N shifts were obtained for 57 out of 59 nonproline sites, ^1^H^α^ shifts at all 62 sites including one Gly residue, ^13^C^α^ shifts at all 61 sites, ^13^C^O^ shifts at 58 out of 61 sites, and ^13^C^β^ shifts at 55 out of 60 sites. To the best of our knowledge, such an extensive set of chemical shifts has not been previously reported for a transiently populated minor protein state. ^1^H and ^13^C CEST experiments (43, 46, 49) were also performed to obtain $\varpi_{I2}$ values for Ala, Ile, Leu, and Val methyl sites (*SI Appendix*, Table S1–S3).

### Folding Intermediate I2 Is Well Ordered but Contains Nonnative Structural Elements and Contacts.

**Fig. 3.**
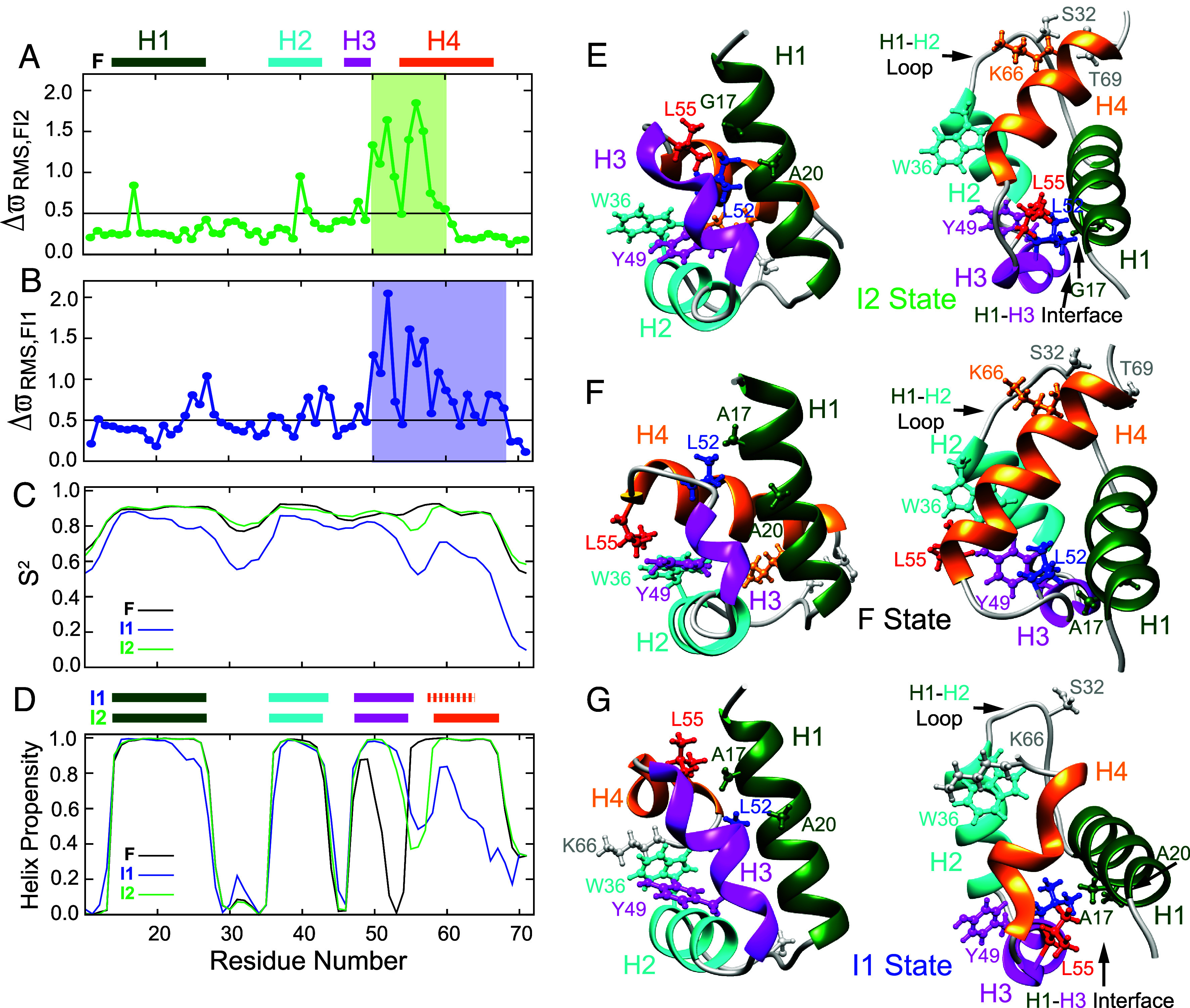
Insights into the structure and dynamics of the I2 state from CEST derived chemicals shifts. Plots of $\Delta \varpi_{RMS,FI2}$ (*A*) and $\Delta \varpi_{RMS,FI1}$ (*B*) as a function of residue. Here, $\Delta \varpi_{RMS,KJ}=\sqrt{\frac{1}{N}\sum_{i=1}^{N}{\left({\Delta \varpi }_{\mathrm{KJ}}^{i}/\varpi_{\mathrm{std}}^{i}\right)}^{2}}$ is calculated for each residue. The summation over *i* extends over all the backbone nuclei (^15^N, ^1^H^N^, ^1^H^α^, ^13^C^α^, ^13^C^O^) and sidechain ^13^C^β^ carbons in the residue for which the $\Delta \varpi_{\mathrm{KJ}}$ values are available, $\varpi_{\mathrm{std}}^{i}$ is the SD in reported chemical shifts (51) for nucleus *i.* In (*A*) and (*B*) the horizontal black line is drawn at $\Delta \varpi_{RMS,FI2/I1}$ = 0.5 ppm. ${\Delta \varpi }_{FI2}$ values (*A*) are from the CEST experiments performed in this study, while ${\Delta \varpi }_{FI1}$ values (*B*) were obtained previously using CPMG experiments (3). (*C* and *D*) Residue specific S^2^/helix propensity values estimated using TALOS-N from the chemical shifts of the F state (black), CEST derived chemical shifts of the I2 state (green), and CPMG derived chemical shifts of the I1 state (blue). Helix boundaries obtained using TALOS-N are shown for the F state above panel *A* (H1 (K14–E27), H2 (W36–I43), H3 (P47–S50), and H4 (L55–Q68)) and for the I2/I1 states above panel (*D*). Helix boundaries in the I2 state are H1 (K14–E27), H2 (W36–I43), H3 (P47–K54), and H4 (K58–Q68) and in the I1 state: H1 (K14–E27), H2 (W36–I44), H3 (P47–L55), and H4 (E57–A64). Two views (orientations) of the A17G FF CEST derived I2 state (*E*), WT FF F state (*F*) [PDB: 1UZC] (33), and WT FF CPMG derived I1 state [PDB: 2KZG] (3) (*G*) structures with key residues shown using the ball and stick representation. In (*E-G*) L55 is colored in red and L52 is colored in blue. All other residues have the same color as the helix in which they are situated or are in gray if they are in loops. Residues from N12 to E70 are shown in the I2 and F state structures (*E* and *F*) while residues from N12 to K66 are shown in the I1 state structure (*G*). In (*E*–*G*) only the lowest energy structure is displayed.

To evaluate, initially qualitatively, the structure of the I2 state, we first compared its chemical shifts to those of the ground state (Fig. 3*A*). It is clear that significant shift changes between the two states ($\Delta \varpi_{RMS,\;FI2}$ > 0.5 ppm) are largely localized to the stretch of residues between S50 and Q60, starting at the C terminal end of H3 and extending into the N-terminal end of H4 in the folded structure (F). The chemical shift changes between F and I1 are more extensive, including some residues at the C terminal end of H1, in addition to the stretch between S50 and Q68 encompassing the whole of H4 (Fig. 3*B*). Residue specific S^2^ values calculated from the backbone chemical shifts (RCI S^2^) (50) provide a measure of the protein backbone flexibility, with S^2^ values > 0.8 indicative of ordered structure, as is observed throughout the F state with the exception of the terminal few residues (Fig. 3*C*). Notably, the S^2^ vs residue correlation for the I2 state is very similar to that of the F conformer (Fig. 3*C*), indicating that I2 is also well ordered. However, I1 is less ordered, with the intervening residues between H1 and H2 as well as H4 less stable (3). Estimates of residue-specific helix propensities derived from backbone chemical shifts establish that I2 also contains four helices, however, a comparison of I2 and F helix propensities shows that H3 extends to K54 in I2 instead of ending at S50 as in F, while H4 starts at K58 in I2 rather than at L55 as in F (Fig. 3*D*).

The extensive set of CEST derived I2 backbone chemical shifts were then used as input into the CS-ROSETTA program (38) to calculate the structure of the A17G FF I2 state (Fig. 3*E*). The CS-ROSETTA protocol converged, with a C^α^ RMSD of 0.8 ± 0.2 Å for the ten lowest energy structures relative to the lowest energy I2 conformer (*SI Appendix*, Fig. S3). In an analogous calculation, serving as a control, and performed using F state chemical shifts for the same sites as for I2, a C^α^ RMSD of 0.9 ± 0.2 Å was obtained; more importantly, a C^α^ RMSD of 1.4 ± 0.2 Å was calculated for the lowest ten energy F state structures relative to the WT FF domain F state structure that was generated using standard NMR (i.e., NOE-based) techniques (33) (*SI Appendix*, Fig. S3). This control calculation provides confidence that the set of CEST-based chemical shifts used in this study is sufficient to define the FF domain I2 structure with CS-ROSETTA.

The CEST derived (A17G FF) I2 state structure along with the (WT FF) F, and (WT FF) I1 state structures are shown in a pair of orientations in Fig. 3 *E*, *F*, and *G*, respectively. As expected, based on the analysis of chemical shifts in Fig. 3 *A*–*D*, the I2 state (Fig. 3*E*) is ordered, compact, and consists of four helices as in the F state (Fig. 3*F*), with H3 elongated at its C-terminal end relative to F. As a consequence of this change in the secondary structure of the H3/H4 region, L55 at the start of H4 and interacting with H2/H3 residues (W36/Y49) in the F state (Fig. 3*F*), is repositioned to within the H3-H4 loop of I2 at the H1/H3 interface (Fig. 3*E*). Thus, in the I2 state L55 occupies the position of L52 in the F state (compare Fig. 3 *E* and *F*). As a consequence, in the I2 state of A17G FF (Fig. 3*E*) the L55 δ1 and δ2 methyl groups are proximal to H^α^ of G17 (H1), while in F of WT FF the L52 δ2 methyl group is proximal to A17 (Fig. 3*F*). Further, repositioning of L52 in the I2 state (Fig. 3*E*) brings it proximal to A20 (H1) and Y49 (H3), in contrast to L52 in F which contacts residue 17. Similar changes in secondary structure and sidechain orientation/packing are seen in the WT I1 state structure (Fig. 3*G*), obtained from CPMG derived chemical shifts (3). In I1, as in I2, H3 is extended at its C terminal end placing L55 in proximity of A17. Unlike I2 and F, however, H4 is largely disordered in I1. Thus, the structure of I2 shares similarities with those of both F and I1. Finally, the loop connecting H1 and H2 (K28 to S35) is rigid in I2 and F but flexible in I1 (Fig. 3*C*). Insight into why this is the case is obtained from the structures which show that this loop docks onto the C-terminal end of H4 in F and I2 (Fig. 3 *E* and *F*) with the sidechain of S32 from the H1-H2 loop in close contact with the sidechains of K66 from H4 and T69 that is adjacent to H4. In contrast, the C-terminal end of H4 is disordered in I1 and consequently unavailable for docking to the H1-H2 loop, which therefore remains flexible.

### Validating the CEST-Derived Structure of I2.

Having calculated the structure of the I2 folding intermediate using a CS-ROSETTA protocol based on an extensive collection of chemical shifts, we next sought to validate it. The distance between the G17 H^α^ and L55 H^δ^ protons is large in the F state (G17 C^α^-L55 C^δ1^/C^δ2^ ~ 15 Å) but is significantly reduced in the I2 state (G17 C^α^-L55 C^δ1^/C^δ2^ ~ 4.5 Å; *SI Appendix*, Fig. S4), despite the fact that the structure in the stretch around G17, E15–A20, does not change significantly between F and I2 (C^α^ RMSD of ~0.3 Å in this region). Focusing on G17, $\left|{\Delta \varpi }_{FI2}\right|$ is small for ^1^H^N^ (0.13 ppm), ^15^N (0.31 ppm), ^13^C^O^ (0 ppm), and ^13^C^α^ (0.22 ppm) nuclei (Fig. 2 *A*–*D*), but large for the two G17 H^α^ sites (0.90 and 0.46 ppm; Fig. 2 *E* and *F*). Further, large $\left|{\Delta \varpi }_{FI2}\right|$ values are also noted for the L55 methyl δ1/δ2 protons (0.44 ppm and 0.65 ppm; Fig. 2 *N* and *P*). Taken together, the large shift changes for G17 H^α^ and L55 H^δ^ protons between F and I2 conformers are consistent with a structural rearrangement involving these pair of residues, with G17-L55 proximal in one conformation (such as I2) and more distal in a second state (such as F), for example.

**Fig. 4.**
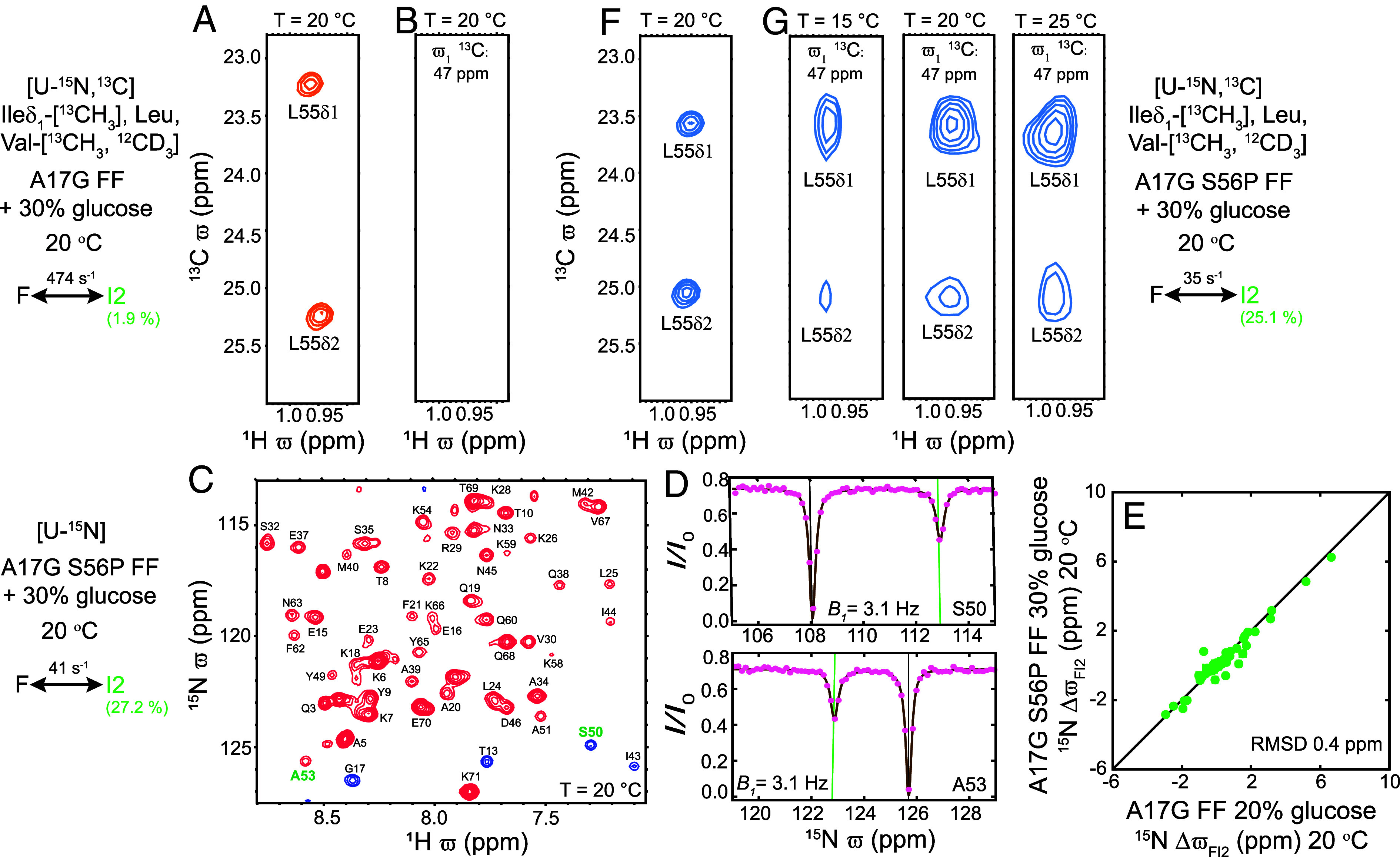
Validating the I2 state structure. (*A*) Selected region of the A17G FF ^1^H-^13^C HSQC map showing L55 ^1^H^δ1^-^13^C^δ1^ and ^1^H^δ2^-^13^C^δ2^ correlations. (*B*) Strip at the G17 ^13^C^α^ chemical shift (47 ppm) from the A17G FF 3D HSQC-NOESY-HSQC spectrum does not contain correlations that would be expected for an NOE between G17 ^1^H^α1/α2^ and L55 ^1^H^δ1/ δ2^ protons (distances < 5 Å). (*C*) Amide ^1^H-^15^N correlation map of [U-^15^N] A17G S56P FF (16.4 T; 20 °C). (*D*) Representative amide ^15^N CEST profiles. Green and black lines are used to indicate $\varpi_{I2}$ and $\varpi_{F}$ values, respectively. (*E*) Correlation between ^15^N CEST derived ${\Delta \varpi }_{FI2}$ values for A17G FF and A17G S56P FF (from *D*). (*F*) Selected region of the A17G S56P FF ^1^H-^13^C HSQC correlation map, highlighting correlations from L55. (*G*) Strips at the G17 ^13^C^α^ chemical shift (47 ppm) from the A17G S56P FF 3D HSQC-NOESY-HSQC spectrum recorded at 15 °C (*k_ex,FI2_* ~ 18.8 ± 0.9 s^−1^; *p_I2_* ~ 20.4 ± 0.5%), 20 °C (*k_ex,FI2_* ~ 35.1 ± 0.9 s^−1^; *p_I2_* ~ 25.1 ± 0.4%), and 25 °C (*k_ex,FI2_* ~ 62.8 ± 2 s^−1^; *p_I2_* ~ 27.6 ± 0.4%) showing NOE correlations between G17 ^1^H^α1/α2^ and L55 ^1^H^δ1/ δ2^ protons. (*A*, *B*, *F*, *G*) The methyl HSQC and NOESY experiments for A17G FF and A17G S56P FF were recorded with samples that were [U-^15^N, ^13^C] at all sites other than the ILV sidechains that were selectively methyl labeled (Ileδ_1_-[^13^CH_3_], Leu, Val-[^13^CH_3_,^12^CD_3_]) (*SI Appendix*, *Materials and Methods*). All the experiments were carried out in 30% [^2^H] glucose, 10% D_2_O buffer. The NOE mixing time was set to 100 ms in all the NOESY experiments. The slight difference in fitted exchange parameters for the F ⇋ I2 interconversion (*C*, *Left*; *G, Right*) reflects the difference in the extent of ^2^H enrichment in the protein samples analyzed.

More conclusive validation of the CEST derived structure of the I2 state can be obtained by recording NOEs, as the small distance between G17 H^α1/α2^ and L55 methyl H^δ1,δ2^ in the I2 state favors cross relaxation between the two sets of protons. However, NOEs between G17 and L55 protons were not observed in a ^13^C-^13^C-^1^H HSQC-NOESY-HSQC dataset recorded with an A17G FF sample at 20 °C using *T_MIX_* = 100 ms (Fig. 4 *A* and *B*) probably because *p_I2_* is only ~2% with rapid exchange to state F (*k_ex,FI2_* = 474 ± 8 s^−1^) where the distance between these protons is large (~15 Å). As S56, which is part of H4 in the F state, repositions to the loop between H3 and H4 in the I2 state structure (Fig. 3*D*), we reasoned that replacing S56 with a proline might increase *p_I2_* by destabilizing F. A high-quality spectrum of A17G S56P FF was obtained (Fig. 4*C*), with only small changes in peak positions relative to A17G FF (^15^N RMSD = 0.2 ppm, excluding positions 55 to 57), and ^15^N CEST data were recorded at 20 °C in 30% glucose buffer to stabilize I2 relative to U and I1 (Fig. 4*D*). The data were well fit to a two-state process of interconversion between F and I2 states (Fig. 4*E*) with *k_ex,FI2_* ~ 41.4 ± 1.3 s^−1^ and *p_I2_* ~ 27.2 ± 0.5% ($\chi_{\mathrm{red}}^{2}$ ~ 1.2), although the exact kinetic parameters depend on the degree of ^2^H enrichment in the protein and the fraction of D_2_O in the solvent. The relatively small differences in ${\Delta \varpi }_{FI2}$ values between A17G and A17G S56P FF samples (Fig. 4*E*, RMSD = 0.4 ppm, excluding residues 55 to 57) indicate that the addition of the proline mutation is unlikely to significantly affect the I2 conformation. Notably, the large increase in *p_I2_* (from ~2% to ~25%, 20 °C) for A17G S56P FF now results in the observation of NOEs between H^α^ protons of G17 and the methyl δ protons of L55 at 15, 20, and 25 °C (*T_MIX_* = 100 ms; Fig. 4*G*), consistent with the fact that L55 and G17 are proximal in the I2 state and in support of the CEST derived I2 structure. It is worth emphasizing that although these NOEs are detected between F state resonances, the NOE enhancement occurs when the molecule samples the I2 state, in a manner analogous to a transferred NOE effect. The origin of these NOEs is made clear from the increase of their intensities with temperature (Fig. 4*G*) which occurs concomitantly with the temperature-dependent increase in *p_I2_* (Fig. 4*G* and *SI Appendix*, Figs S4 and S5). In contrast, if the NOEs were to arise from magnetization transfer exclusively within the F state an opposite temperature dependence would be predicted, since in the macromolecular limit the NOE scales directly with rotational correlation time, which becomes smaller with increase in temperature. Finally, the observed NOEs do not arise from transfer within the I1 conformer as a three-state analysis of the A17G S56P FF ^15^N CEST data establishes that *p_I1_ <* 2% at 20 °C (*SI Appendix*, Fig. S6), too low of a population to give rise to observable NOE peaks (Fig. 4*B*). The excellent correlation between the amide ^15^N ${\Delta \varpi }_{FI2}$ values of A17G and WT FF [RMSD ~ 1.1 ppm; (31, 32)] suggests that the I2 state structure derived here is a good mimic of the I2 conformer in WT FF. However, it is worth noting that the A17G mutation can lead to differences between the two structures at the level of sidechain packing.

### CEST Derived Urea *m*-Values Provide Additional Insights into the FF Domain Folding Mechanism.

**Fig. 5.**
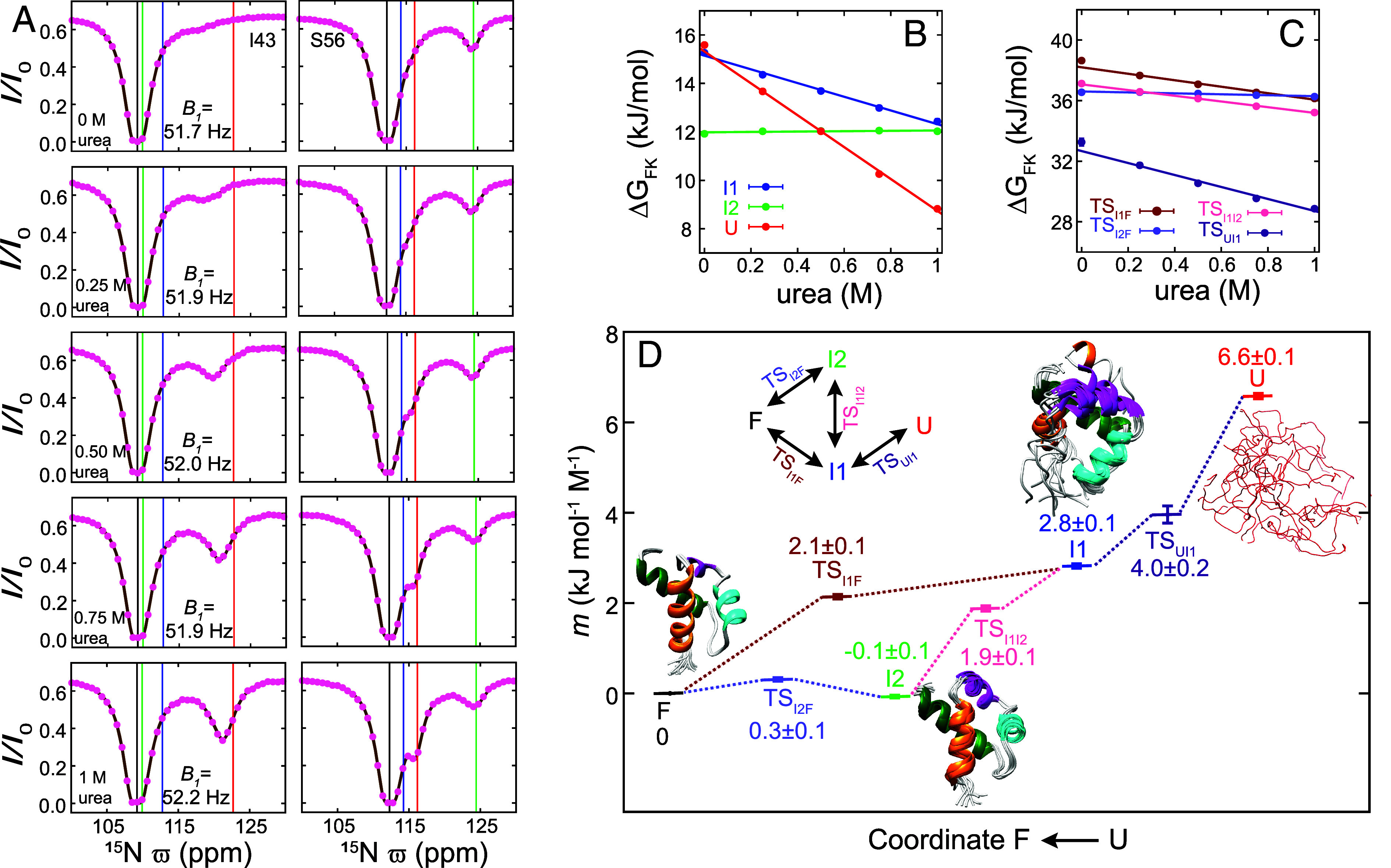
*m*-values describe how the compaction of A17G FF evolves along the folding trajectory. (*A*) Representative amide ^15^N CEST profiles from I43 and S56 in A17G FF (16.4 T; *T_EX_* = 400 ms; 2.5 °C) recorded with varying amounts of urea. Experimental data are shown with pink circles while the continuous brown line in each panel is calculated from the best-fit parameters. The vertical black, blue, green, and red lines correspond to the fitted $\varpi_{F}$, $\varpi_{I1}$, $\varpi_{I2}$, and $\varpi_{U}$ values, respectively. (*B*) Variation of $\Delta G_{FI1}$, $\Delta G_{FI2}$, and $\Delta G_{\mathrm{FU}}$ as a function of urea concentration. The *m*-value for state K (*m_K_*) is obtained from the slope of the urea concentration dependence of $\Delta G_{\mathrm{FK}}=-RTln\left(p_{K}/p_{F}\right)$, with $p_{K}$ and $p_{F}$ obtained from the analysis of the amide ^15^N CEST profiles recorded at different urea concentrations, as illustrated in (*A*). (*C*) Variation of $\Delta G_{FTSI1F}$, $\Delta G_{FTSI2F}$, $\Delta G_{FTSI1I2}$, and $\Delta G_{FTSUI1}$ as a function of urea concentration, where $\Delta G_{\mathrm{FTSKJ}}$ is the free energy difference between TS_KJ_ and the F state. The *m*-value for transition state TS_KL_ (*m_TSKL_*) is obtained from the slope of the urea concentration dependence of $\Delta G_{\mathrm{FTSKL}}=-RTln\left(p_{K}/p_{F}\right)-RTln\left(k_{\mathrm{KL}}/C\right)$, where $p_{K}$, $p_{F}$ and $k_{\mathrm{KL}}$ are derived from the analysis of ^15^N CEST profiles recorded as a function of urea concentration. The constant *C* is set to be $10^{7}$ s^−1^ and does not affect the value of *m_TSKL_*. Best-fit four-state exchange parameters obtained at various urea concentrations are listed in *SI Appendix*, Table S4. (*D*) Urea *m*-values obtained from (*B*) and (*C*) at various points along the folding landscape of A17G FF. Ten structures (W11–K71) of the F state (WT FF), the I1 state (WT FF), and the I2 state (A17G FF) are shown. In the case of the I1 state, residues corresponding to the disordered H4 helix have been added for the purpose of illustration.

With atomic resolution structures of the F, I1, and I2 states now available, we next sought to quantify the structural rearrangements that occur during their interconversion. For example, does the FF domain undergo extensive unfolding when it interconverts between compact F and I2 states to facilitate breakage of nonnative interactions and formation of native contacts (I2 to F) or vice versa (F to I2)? The urea *m*-value is a measure of how the free energy differences between states vary with urea concentration (45), with the *m*-value of state J with respect to the folded state (F) defined as $m_{J}=-\frac{d\Delta G_{\mathrm{FJ}}}{d\left[urea\right]}$, where $\left[urea\right]$ is the urea concentration, $\Delta G_{\mathrm{FJ}}=G_{J}-G_{F}$, and $G_{J}$ is the Gibbs free energy of state J. States with greater solvent-exposed surface areas have more urea binding sites and consequently higher urea *m*-values, so that the urea *m*-value is a reporter for the compactness of the interconverting states, including the transition states involved (note that J can be a transition state) (52). For example, a U state that is completely disordered and expanded would have a higher *m*-value than other, partially folded or completely folded, conformers. To obtain *m*-values for the three minor states and four transition states of the A17G FF folding trajectory, a series of ^15^N CEST datasets (Fig. 5*A*) was recorded at 2.5 °C using A17G FF samples prepared with varying amounts of urea (from 0 to 1 M). The resulting CEST profiles were fit to the four-state exchange model of Fig. 1*B* to obtain exchange parameters at the different urea concentrations (*SI Appendix*, Table S4). As discussed in *SI Appendix*, *m*-values for the three minor states were obtained from the urea dependencies of their populations, while the transition state *m*-values were calculated from the urea dependencies of populations and rate constants (Fig. 5 *B* and *C*). Recall that all *m*-values reported here are with respect to the F state. As expected, among the three minor states, U has the highest *m*-value (6.6 ± 0.1 kJ mol^−1^ M^−1^) as it is disordered, I1 the second-highest *m*-value (2.8 ± 0.1 kJ mol^−1^ M^−1^), consistent with it being fairly structured but with H4 disordered, while *m*_*I2*_ (−0.1 ± 0.1 kJ mol^−1^ M^−1^) is indistinguishable from *m_F_* (0 kJ mol^−1^ M^−1^ by definition), consistent with an I2 state structure that is as compact as the F state. To some extent these *m*-values can be rationalized by visually inspecting the amide ^15^N CEST profiles recorded at different urea concentrations (Fig. 5*A*). In the ^15^N CEST profile of S56 the size of the I2 state dip at $\varpi_{I2}$ (near the green line) does not change with urea concentration consistent with little change in *p_I2_* and, hence, *m*_*I2*_ ~0 kJ mol^−1^ M^−1^. On the other hand, the dip that results from the (rapid) I1-U interconversion, located between $\varpi_{I1}$ (blue line) and $\varpi_{U}$ (red line) becomes more prominent and moves toward $\varpi_{U}$ as the urea concentration increases, as the population of the U state increases more rapidly with urea than the population of I1, a result consistent with *m*_*U*_ > *m*_*I1*_. Transition state *m*-values, *m*_*TSAB*,_ can also be interpreted in terms of the compactness of the respective transition state (TS_AB_), in this case connecting states A and B. For example, *m*_*TSUI1*_ (4.0 ± 0.2 kJ mol^−1^ M^−1^) lies in between *m*_*U*_ (6.6 ± 0.1 kJ mol^−1^ M^−1^) and *m*_*I1*_ (2.8 ± 0.1 kJ mol^−1^ M^−1^), establishing that TS_UI1_ is more compact than U but less compact than I1. Considering the two folding trajectories of the four-state model of Fig. 1*B* used to analyze the data, *m*_*U*_ > *m*_*TSUI1*_ > *m*_*I1*_ > *m*_*TSI1F*_ > *m*_*F*_ and *m*_*U*_ > *m*_*TSUI1*_ > *m*_*I1*_ > *m*_*TSI1I2*_ > *m*_*I2*_ (Fig. 5*D*), it is clear that the FF domain progressively becomes more compact as the folding reaction proceeds from U to I1 to F and from U to I1 to I2. However, *m*_*TSI2F*_ (0.3 ± 0.1 kJ mol^−1^ M^−1^) is only slightly larger than *m*_*F*_ ~ *m*_*I2*_ (~0 kJ mol^−1^ M^−1^), establishing that interconversion from the compact I2 state to F, involving changes in secondary structure and breaking of nonnative interactions, also proceeds via a compact transition state TS_I2F_ that is only slightly larger than F and I2 but significantly more compact than both TS_I1F_ (*m*_*TSI1F*_ = 2.1 ± 0.1 kJ mol^−1^ M^−1^) and the I1 folding intermediate (*m_I1_* = 2.8 ± 0.1 kJ mol^−1^ M^−1^). The profile of urea *m*-values along the A17G FF folding trajectory reported here agrees, generally, with that published previously (4); however, there are some differences. In the present work, measurements were carried out at 2.5 °C, as opposed to 25 °C, and it is known that urea *m*-values decrease with increasing temperature (53). In addition, the previous analysis of FF domain folding data did not consider the I2 state (i.e., only included F, I1, and U), yet it is now clear that I1 can either convert to F directly or via I2. It is likely that neglect of this second I1 to F pathway through I2 leads to some of variations in *m*-values in the two studies.

## Discussion

Herein we present a four-state model describing the folding pathway of the FF domain, including atomic resolution structural models of the two intermediate states, I1 and I2, that are formed during this process. Starting from the U state there is rapid collapse on the ~100 µs timescale, forming I1 that contains H1, H2, and an elongated H3, with stabilization of these elements occurring through a network of nonnative (and native) contacts. State I1 (*m_I1_* = 2.8 ± 0.1 kJ mol^−1^ M^−1^) is more compact than state U (*m_U_* = 6.6 ± 0.1 kJ mol^−1^ M^−1^), but more loosely packed compared to state F. Maximizing hydrophobic interactions presumably is the driving force behind the rapid collapse from U to I1, resulting in the formation of nonnative structural elements in I1. The I1 intermediate is then able to fold to F either in a single step that involves both the breaking of nonnative interactions and the formation of H4 or in a two-step manner via I2 that first involves formation of H4 followed by the breaking of nonnative interactions when I2 converts to F. From the fitted kinetic rates it is clear that the barrier for the interconversion between native and nonnative structural features (F ⇋ I1, F ⇋ I2) is higher than the barrier for the order/disorder transition (I1 ⇋ U, I2 ⇋ I1). Slow rates associated with “fixing” nonnative interactions to form the folded state have also been observed in a comparative study of the WT and L24A FF domains (24, 35). The I1 state of the L24A FF variant was shown to be stabilized by an increased number of nonnative contacts relative to the corresponding WT intermediate, accounting for the approximate ten-fold slower I1 to F folding rate for the mutant domain (24). The fact that formation of the F state from I2 can proceed without significant changes in compaction of TS_I2F_ is notable, considering the significant structural rearrangements that must occur. A similar situation has been observed previously in studies of the L99A cavity mutant of T4 lysozyme. In this case, F114 that is at the junction of two helices and exposed to solvent in the major state becomes buried in the core of the protein in a transiently populated minor state (23). Just as in the case of the I2 ⇋ F FF domain interconversion, computations show that F114 is able to swing into the core of protein without any largescale expansion as two neighboring helices transiently move apart by a few Å (54).

Additional insight into the accumulation of structure along the folding trajectory, focusing on transition states, may be obtained via Φ-value analysis (27). An extensive set of experiments on the WT FF domain and associated mutants (35), analyzed assuming a simple F ⇋ U folding reaction, shows that only a small part of the protein that includes the end of H1, the beginning of H2 and intervening loop residues are structured in the rate-limiting transition state. The transition state described by this analysis largely corresponds to TS_I1F_ (3), as it is more stable than TS_I2F_ under the conditions used to perform the Φ-value experiments (2 and 6 M urea). Thus, only regions of I1 that are native-like are conserved in TS_I1F_, while the start of H1 and the H3-H4 loop that form nonnative contacts and H3 that is elongated in I1 are not preserved. Helix H4 is not formed in TS_I1F_ but tertiary interactions between H4 residues and the protein core are beginning to emerge (35). Data from TS_I2F_, TS_I1I2_, and TS_UI1_ are not available from this Φ-value analysis. However, Φ-values for all these transition-states can now be measured because ^15^N CEST data recorded using various FF mutants (for Φ-value analysis) can be analyzed (separately) to obtain four-state exchange parameters.

The I2 state structure determined in this study is as compact and as ordered as the F state. We wondered, therefore, whether I2-like structures might serve as ground states of other FF domains, and, if this is the case, whether such a structure could be functionally relevant. Indeed, in the native state structure of the FF1 domain from human p190-A Rho GAP (RhoGAP-FF1) helix H3 is elongated relative to what is observed in the canonical FF native state (55), and the equivalent of L55 in the canonical FF domain (L311) makes a contact with the equivalent of A17 (A273) in H1, as in the I2 state structure (*SI Appendix*, Fig. S7). Interestingly Y308 (L52 here) of RhoGAP-FF1 that becomes phosphorylated to inhibit an interaction with the transcription factor TFII-I (55, 56) is buried in the core of the domain and cannot be accessed by the kinase. Based on the observation that phosphorylation occurs above 310 K, a temperature at which the amide ^1^H-^15^N correlation map becomes less well-dispersed, it had been suggested that RhoGAP-FF1 must unfold for Y308 to be phosphorylated (55, 57). However, the melting point of RhoGAPFF1 is 325 K, somewhat higher than 310 K at which kinase activity has been observed. Building on our current understanding of FF dynamics where states I1 and I2 rapidly interconvert with each other, it may well be the case that rather than phosphorylating the U state the kinase phosphorylates RhoGAP-FF1 in the I1 state where Y308 is accessible because H4 is disordered (*SI Appendix*, Fig. S7). The observation that the native state of RhoGAP-FF1 has the same conformation as the I2 folding intermediate of the FF domain from human HYPA/FBP11 suggests that compact, ordered folding intermediates can be repurposed by nature in different, but important functional roles in structurally related molecules. Another interesting take-away that emerges, in this case from the structures of both the I1 and I2 states, is that nonnative helical extensions are found in both minor conformers (helix H3 in both cases), as is also observed in the case of the L99A cavity mutant of T4 lysozyme (23) where helices F and G reposition to form a single long helical structure in an excited state. Albeit only a few examples, this may suggest that intermediates use helix elongation as a mechanism for stabilization and that, therefore, elongated helices may be a feature in other transiently populated minor states as well.

Previous NMR studies have generated structures of single intermediates along reaction pathways (3, 18, 20–26). We show here that CEST is a particularly powerful method for more extensive structural studies, involving pathways with at least two intermediates. In the strategy proposed in this study, we have used peak positions and linewidths in ^15^N CEST profiles to advance a folding model, in this case four-state (32), and then a combination of single mutations and/or additives to isolate one of the exchange reactions, for example, F ⇋ I2, so that state I2 can subsequently be studied in detail through a comprehensive set of ^1^H, ^15^N, and ^13^C CEST experiments. In general, the isolated two-state exchange component, F ⇋ X, of the more complex four-site model, can be identified by comparing CEST-derived X state ^15^N chemical shifts with those obtained from fits of ^15^N CEST profiles in the four-state exchanging system. Although in this case, the I1 structure was determined previously (3), had this not been the case we could have chosen the A39G FF domain dissolved in 10% TFE buffer, as the exchange reaction reduces to a two state F ⇋ I1 process, with *p_I1_* ~ 9% (1 °C) (32). Since the CEST experiment is not restricted to folding studies, but can be used to explore other biomolecular process, it is likely that the approach we have utilized here to characterize the folding landscape of the FF domain at atomic resolution will be applicable to other exchanging protein systems as well.

## Materials and Methods

The CEST datasets were recorded using previously published sequences (30, 42, 43, 46–49) on protein samples that were isotopically (^15^N/^13^C/^2^H) enriched at the appropriate sites. FF samples with the desired isotopic enrichment patterns were prepared by overexpressing the protein in *E coli* BL21(DE3) cells grown in suitable M9 media (58, 59). To obtain the exchange parameters and minor-state chemical shifts, CEST datasets were analyzed using the program *ChemEx* (60). CS-ROSETTA (38) was used to calculate structures of A17G FF in the I2 state from the CEST derived $\varpi_{I2}$ values. All the CEST NMR experiments were performed on a 16.4 T (700 MHz) Bruker Avance III HD spectrometer equipped with a cryogenically cooled triple resonance probe. *SI Appendix* *c*ontains details regarding protein expression and purification, NMR experiments, data analysis, and brief discussions about CEST and *m*-value analyses.

## Supplementary Material

Appendix 01 (PDF)

Dataset S01 (GZ)

## Data Availability

The CEST derived chemical shifts of A17G FF in the I2 state (*SI Appendix*, Table S3) and the coordinates of the ten lowest energy A17G FF I2 state structures (A17GFF_I2_lowest10.pdb.gz) are included in the supporting information.
