# Supplementary material for: Mapping the FF domain folding pathway via structures of transiently populated folding intermediates
Source: Proc Natl Acad Sci U S A. 2024 Dec 4;121(50):e2416682121. doi: 10.1073/pnas.2416682121 (PMC11648910; doi:10.1073/pnas.2416682121)
Supplement: Supplementary file 1 — Appendix 01 (PDF) [file pnas.2416682121.sapp.pdf]

Supporting Information

Mapping the FF domain folding pathway via structures of transiently populated folding intermediates

This PDF file includes:

Supplementary text

Figures S1 to S7

Tables S1 to S4

Legend for supplementary file A17GFF_I2_lowest10.pdb.

SI References

Chemical Exchange Saturation Transfer

Consider a simple two-state reaction, $F \leftrightharpoons U$, where states F and U are in slow exchange on the NMR chemical shift timescale, with $p_F \gg p_U$ (1). A set of spectra is acquired with a weak radio frequency (B_1) pulse applied at a given frequency for an exchange time, T_{EX} (one spectrum for each position of the B_1 field), and the effect of the B_1 irradiation on the 'visible' major state spectrum is monitored (2-4). A plot of the normalised intensity of a peak derived from the visible (F) state (I/I_0) vs ϖ_{RF} will have two dips, one at the chemical shift (ppm) of the major state, ϖ_F, and, importantly, one at the chemical shift of the minor state ϖ_U. Here ϖ_{RF} is the offset (ppm) at which the B_1 field is applied. The exchange parameters ($k_{ex,FU}$, p_U), major/minor state chemical shifts, major state longitudinal relaxation rate, and major and minor state transverse-relaxation rates can all be obtained by analysing CEST profiles recorded with two different B_1 values (5, 6). Exchange occurring at rates spanning the ~10 to ~10,000 s^{-1} range can be studied using amide ^{15}N CEST experiments, although as exchange becomes fast only a single dip will be observed in each CEST profile at a frequency of $\sim\varpi_F$, and its shape could be asymmetric – slightly tilted towards the position of the minor state (6-9). Interestingly, introduction of a third state I that is in rapid exchange with U, $F \leftrightharpoons I \leftrightharpoons U$ such that $p_F \gg p_U \gg p_I$ and $k_{ex,IU} \gg k_{ex,FI}$ results in a CEST intensity profile with two dips, one corresponding to state F and one arising largely from state U, but with the minor U state dip broadened and moved from ϖ_U towards ϖ_I (Fig. S1). Recently we have shown that the broadening of the 'U' state dip can be used to detect other minor states, such as I in this example, that otherwise would have escaped detection by other methods (10, 11).

Materials and Methods

NMR Samples. Isotopically enriched A17G FF and A17G S56P FF domains were overexpressed in *E coli* BL21(DE3) cells transformed with the appropriate plasmid and grown in the appropriate M9 media (12, 13). As all the samples used in this study were uniformly ^{15}N enriched, the M9 media used to grow the cells consisted of 1g/L $^{15}NH_4Cl$ as the sole nitrogen source. To prepare [U-^{15}N] labelled protein 5 g/L of glucose was added as the sole carbon source. [U-^{15}N, ^{13}C] labelled protein was expressed in cells grown in M9 media consisting of 3 g/L of [$^{13}C_6$] glucose as the sole carbon source. [U-^{15}N], $^{13}C^{\alpha}$ labelled protein was expressed in cells grown in M9 media consisting of 3 g/L of [2-^{13}C] glucose as the sole carbon source (12, 14). [U-^{15}N, ^{13}C] 50% ^{2}H labelled protein was expressed in cells grown in 60% D_2O M9 media with 3 g/L of [$^{13}C_6$] glucose as the sole carbon source (15, 16). The NOESY experiments described in the text were measured on a sample that is [U-^{15}N, ^{13}C] enriched everywhere except for the side-chains of Ile, Leu and Val that are [Ileδ1 - $^{13}CH_3$, ^{2}H; Leu, Val - $^{13}CH_3/^{12}CD_3$, ^{2}H]. This sample was prepared by growing cells initially in M9 media with 3 g/L of [$^{13}C_6$] glucose as the sole carbon source. One hour prior to induction of protein expression 50 mg/L 2-keto-3-d$_2$-4-^{13}C butyrate and 100 mg/L 2-keto-3-methyl-d$_3$-3-d$_1$-4-^{13}C-butyrate were added to the media (13). The overexpressed protein with the desired labelling was purified

from the *E coli* cells using a two-step procedure consisting of a cation exchange chromatography step followed by a size exclusion chromatography step, as described previously (16).

NMR Samples. See *SI Appendix*, Table S1 for a list of all samples used in the present study.

NMR Experiments. All CEST and NOESY experiments were recorded on a 700 MHz Bruker Avance III HD spectrometer equipped with a triple-resonance cryogenically cooled probe with a Z axis gradient. The assignment experiments were performed on a 500 MHz Bruker NEO spectrometer equipped with a room temperature triple resonance Z-gradient probe.

In this study we have used CEST rather than CPMG experiments to obtain ϖ_{I2} values because i) ^{13}C CEST experiments can be performed using uniformly ^{13}C enriched samples (17, 18), unlike the case for CPMG studies (12, 14), resulting in a more extensive set of I2 state chemical shifts, ii) accurate exchange parameters can be obtained from CEST experiments performed at a single B_0 field (4, 5) and iii) ϖ_{I2} values are directly obtained from the analysis of CEST profiles, while only the absolute value of $\Delta\varpi$ ($|\Delta\varpi|$) is available from the analysis of CPMG data. Thus, additional experiments, sometimes at multiple B_0 field strengths, must be performed to obtain the sign of $\Delta\varpi$ and reconstruct the minor state spectrum from CPMG data (19-21).

All CEST experiments were carried out in a pseudo 3D manner (B_1 offset in the third dimension), with CEST profiles generated by quantifying peak intensities from two-dimensional correlation maps. Amide ^{15}N ϖ_{I2} shifts were obtained from ^{15}N CEST datasets recorded on a [U-^{15}N, ^{13}C] A17G FF sample (sample 1; *SI Appendix*, Table S1) using the standard ^{15}N CEST experiment (4). Amide ^{1}H^{N} ϖ_{I2} shifts were obtained by recording ^{1}H^{N} CEST datasets with suppression of NOE-based dips (22, 23) ([U-^{15}N, ^{13}C] A17G FF sample; sample 1). ^{13}C^{O} ϖ_{I2} shifts were obtained from ^{13}C^{O} CEST datasets recorded on a [U-^{15}N, ^{13}C] A17G FF sample (sample 1), either using HN(CO)-type or H(N)CO-type sequences in which CEST profiles were obtained by quantifying intensities from ^{15}N-^{1}H^{N} or ^{13}C^{O}(i-1)-^{1}H^{N}(i) correlation maps, respectively (24). ^{13}C$^{\alpha}$ ϖ_{I2} shifts were measured using four different CEST-based experiments: i) ^{13}C$^{\alpha}$ CEST via an (HACACO)NH scheme recorded on a [U-^{15}N, ^{13}C] A17G FF sample (sample 1), with peak intensities quantified from ^{15}N-^{1}H^{N} correlation maps (25); ii) ^{13}C$^{\alpha}$ CEST based on quantification of CT-^{13}C$^{\alpha}$-^{1}H$^{\alpha}$ correlation maps (17) and recorded on a 100% D_2O [U-^{15}N, ^{13}C] A17G FF sample (sample 2); iii) ^{13}C$^{\alpha}$ CEST using a 100% D_2O [U-^{15}N] ^{13}C$^{\alpha}$ A17G FF sample (sample 3), with peak quantification from ^{13}C$^{\alpha}$-^{1}H$^{\alpha}$ correlation maps (17); iv) Gly-optimized ^{13}C$^{\alpha}$ CEST recorded on a 100% D_2O [U-^{15}N, ^{13}C] A17G FF sample (sample 2) with peak intensities quantified from ^{13}C$^{\alpha}$-^{1}H$^{\alpha}$ correlation maps (17). ^{1}H$^{\alpha}$ ϖ_{I2} shifts were obtained from four different CEST experiments all designed to supress NOE dips arising from interactions with remote protons: i) (HACACO)NH-^{1}H$^{\alpha}$ CEST using a [U-^{15}N, ^{13}C] A17G FF sample (sample 1), quantifying peak intensities from a series of ^{15}N-^{1}H^{N} correlation maps (26); ii) CT-^{13}C$^{\alpha}$-^{1}H$^{\alpha}$ ^{1}H$^{\alpha}$ CEST (17, 22) using a 100% D_2O [U-^{15}N, ^{13}C] A17G FF sample (sample 2) ; iii) ^{1}H$^{\alpha}$ CEST but using a 100% D_2O [U-^{15}N] ^{13}C$^{\alpha}$ A17G FF sample (sample 3) with peak quantification from ^{13}C$^{\alpha}$-^{1}H$^{\alpha}$ correlation

maps (17, 22); iv) Gly-optimized $^{1}H^{\alpha}$ CEST using a 100% D_2O [U-^{15}N, ^{13}C] 50% ^{2}H A17G FF sample (sample 4), with peak quantification from a set of $^{13}C^{\alpha}$-$^{1}H^{\alpha}$ correlation maps (15). The $^{13}C^{\alpha}$ A17G FF sample (sample 3) is crucial to study exchange at $^{1}H^{\alpha}$ and $^{13}C^{\alpha}$ sites when the $^{13}C^{\alpha}$ and $^{13}C^{\beta}$ carbons are strongly coupled, as is the case for S56, for example. $^{13}C^{\beta}$ ϖ_{I2} shifts were obtained from three different CEST experiments: i) (HBCBCACO)NH-$^{13}C^{\beta}$ CEST using a [U-^{15}N, ^{13}C] A17G FF sample (sample 1) and quantifying peak intensities from ^{15}N-$^{1}H^{N}$ correlation maps (25); ii) Ser-optimized $^{13}C^{\beta}$ CEST using a 100% D_2O [U-^{15}N, ^{13}C] A17G FF sample (sample 2) and quantifying peak intensities from CT-$^{13}C^{\beta}$-$^{1}H^{\beta}$ correlation maps (17); iii) As in ii) but using a Thr-optimized pulse scheme for Thr residues (17); Methyl ^{13}C (including Ala $^{13}C^{\beta}$) ϖ_{I2} shifts were obtained from methyl ^{13}C CEST experiments recorded on a [U-^{15}N, ^{13}C] A17G FF sample (sample 1) (17, 27). Methyl ^{1}H ϖ_{I2} shifts were obtained using a methyl ^{1}H CEST experiment that supresses dips in CEST profiles arising from dipolar interactions with remote protons (22) (100% D_2O [U-^{15}N, ^{13}C] A17G FF sample; sample 2). In both methyl ^{13}C and ^{1}H CEST experiments peak intensities were quantified from CT-^{13}C-^{1}H correlation maps. Additional details can be found in *SI Appendix*, Tables S1 and S2.

Amide ^{15}N CEST (methyl ^{13}C D-CEST (9, 28)) experiments were used to obtain two-state (F $\leftrightharpoons$ I2) exchange parameters in A17G S56P FF samples dissolved in 10 (100) % D_2O buffer; these samples were used for measurement of NOEs to confirm the I2 state structure. To estimate p_{I1} at 20 °C for the A17G S56P FF sample (sample 13) used to perform the NOESY experiments (Fig. 4), amide ^{15}N CEST datasets were recorded with four different B_1 (T_{EX}) values: 3.2 Hz (400 ms), 53.6 Hz (250 ms), 107.3 Hz (175 ms), and 214.5 Hz (175 ms).

NOEs between G17 H^{α} and L55 H^{δ} were recorded using 3D HSQC-NOESY-HSQC or HSQC-NOESY-HMQC experiments, [Gly $^{13}C^{\alpha}$ (t_1), methyl ^{13}C (t_2), methyl ^{1}H (t_3)], using A17G FF (sample 5) or A17G S56P FF (sample 13,14) samples that are [U-^{15}N, ^{13}C] enriched at all positions except for the side-chains of Ile, Leu and Val that are [U-^{15}N], [Ileδ1 - $^{13}CH_3$, ^{2}H; Leu, Val - $^{13}CH_3$/$^{12}CD_3$, ^{2}H]. The mixing time was set to 100 ms in all the experiments.

Chemical shift assignments of A17G S56P FF were obtained starting from previous assignments for A17G FF, and completed by analysis of standard HNCA, HNCO, HNCACO, HNCACB, (H)C(CO)NH-TOCSY, and H(C)(CO)NH-TOCSY datasets (1, 29-31) at 15 °C (11.7 T).

NMR Data processing and analysis. NMR data were processed using NMRPipe (32), visualised and assigned using SPARKY (33, 34), with peak intensities quantified using PINT (35). TALOS-N (36) was used to analyse the chemical shifts and obtain residue specific helix propensities, residue specific S^2 values (37), and helix boundaries for the various conformational states of A17G FF. The program *ChemEx* (38) that numerically integrates the Bloch-McConnell equations (39) was used to obtain the best-fit exchange parameters from the CEST data.

Chemical shifts for the I2 folding intermediate were obtained by analysing A17G FF CEST data in a two-state manner with global fitting parameters, $k_{ex,FI2}$ and p_{I2}, and residue specific fitting parameters $R_{2,F}$, $R_{1,F}$, $R_{2,I2}$, ϖ_F and $\Delta\varpi_{FI2}$. In all analyses R_1 values were assumed to be the same for all conformers (4, 5). In the analysis of some of the CEST data the exchange parameters were fixed to those obtained from amide ^{15}N or $^{13}C^{\alpha}$ CEST data (See Table S2). To extract the best-fit four-state (F, I1, I2 and U) exchange parameters from analysis of the A17G FF amide ^{15}N CEST profiles, the four-state model in Figure 1B was used, subject to the constraint $R_{2,F} = R_{2,I1} = R_{2,I2} = 2R_{2,U}$ (11). In this case, the global fitting parameters were $k_{ex,FI1}$, $k_{ex,FI2}$, $k_{ex,I1I2}$, $k_{ex,FU}$, p_{I1}, p_{I2} and p_U ($k_{ex,I2U} = k_{ex,FU} = 0$ s^{-1} (11)), with residue specific fitting parameters of: $R_{2,F}$, $R_{1,F}$, ϖ_F, $\Delta\varpi_{FI1}$, $\Delta\varpi_{FI2}$ and $\Delta\varpi_{FU}$. To obtain urea m-values A17G FF ^{15}N CEST data recorded on samples prepared with five different concentrations of urea were analysed under the assumption that $\Delta\varpi_{FI1}$, $\Delta\varpi_{FI2}$ and $\Delta\varpi_{FU}$ are independent of urea concentration with $\Delta\varpi_{FI1}$, $\Delta\varpi_{FI2}$ and $\Delta\varpi_{FU}$ values initialised to those obtained previously from the four-state analysis of the A39G FF data (11).

To extract the best-fit three-state (F, I1, and I2) exchange parameters from the A17G S56P FF amide ^{15}N CEST data, the three-state triangular model in which all three states interconvert with each other was fit to CEST data subject to the constraint $R_{2,F} = R_{2,I1} = R_{2,I2}$. Global (residue specific) fitting parameters were $k_{ex,FI1}$, $k_{ex,FI2}$, $k_{ex,I1I2}$, p_{I1} and p_{I2} ($R_{2,F}$, $R_{1,F}$, ϖ_F, $\Delta\varpi_{FI1}$ and $\Delta\varpi_{FI2}$), and $\Delta\varpi_{FI1}$ and $\Delta\varpi_{FI2}$ were initialised to the values previously determined from the four-state analysis of the A39G FF ^{15}N CEST data (11). Two-state analysis of the A17G S56P FF CEST data followed as for A17G FF, with $k_{ex,FI2}$ and p_{I2} ($R_{2,F}$, $R_{1,F}$, $R_{2,I2}$, ϖ_F and $\Delta\varpi_{FI2}$) global (residue specific) fitting parameters. Bootstrap or Monte Carlo procedures (100 trials) were used to estimate the uncertainties in all the fitted exchange parameters (40).

Structure calculations. The CS-ROSETTA (41) protocol was used to calculate the structural ensemble of the A17G FF I2 state solely from chemical shifts (41-43). 10,000 structures were calculated and the (rescaled) energy versus C^{α} RMSD to the lowest energy structure clearly shows that calculation has converged, with an RMSD of 0.8 ± 0.2 Å for the lowest energy structure to the 10 lowest energy structures (Fig. S3). As a control the same structure calculation procedure was repeated using F state shifts for the same sites that the I2 state shifts are available. This calculation also converged; the 10 lowest energy structures have a C^{α} RMSD of 0.9 ± 0.2 Å to lowest energy structure and a C^{α} RMSD of 1.4 ± 0.2 Å to the lowest energy F state structure of WT FF obtained by conventional NMR based methods (44) (Fig. S3). Residues W11 to Q68 ($S^2 \geq 0.65$) were used for the RMSD calculations. Some of the CS-ROSETTA calculations were performed on NMRbox (45). Structures were visualised and analysed using UCSF Chimera (46).

Extracting urea *m*-values. Starting from the reaction $F \leftrightharpoons K$, where F and K are a pair of exchanging states (potentially of a more complex exchange pathway) it follows directly that $\Delta G_{FK} = G_K - G_F = -RTln(\frac{p_K}{p_F})$, where R and T are the gas constant and the absolute temperature, respectively. Writing

$\Delta G_{FK}(urea) = \Delta G_{FK}(0M\ urea) - m_{FK}[urea]$, it becomes clear that $\frac{d\Delta G_{FK}}{d[urea]} = -m_{FK}$ and, therefore, the slope of the $\Delta G_{FK} = -RTln(\frac{p_K}{p_F})$ vs $[urea]$ plot is $-m_{FK}$. Further, it is straightforward to show that for the reaction $F \leftrightarrows L \leftrightarrows \cdots \leftrightarrows K$ $\Delta G_{FK} = -RTln(\frac{p_K}{p_F})$ as well, so that the slope of the ΔG_{FK}vs $[urea]$ plot is also $-m_{FK}$. In a similar manner the urea m-value of the transition state connecting states K and L (TS_{KL}), for example, can be obtained by defining $\Delta G_{FTSKL} = G_{TSKL} - G_F$, where G_{TSKL} is the free energy of the transition state. Writing $\Delta G_{FTSKL} = (G_{TSKL} - G_K) - (G_F - G_K)$ and noting that $k_{kl} = Cexp(-\frac{G_{TSKL}-G_K}{RT})$, where k_{KL} is the forward rate constant for the reaction $K \leftrightarrows L$, and C is a constant, it follows that $\Delta G_{FTSKL} = -RTln\left(\frac{k_{KL}}{C}\right) - RTln(\frac{p_K}{p_F})$. Noting that $\Delta G_{FTSKL}(urea) = \Delta G_{FTSKL}(0M\ urea) - m_{TSKL}[urea]$, it follows that the slope of the $\Delta G_{FTSKL} = -RTln\left(\frac{k_{LK}}{C}\right) - RTln(\frac{p_L}{p_F}))$ vs $[urea]$ plot is $-m_{TSKL}$. While C was set to be 10^7 s^{-1} in this study, the value of C does not affect the extracted transition state m value.

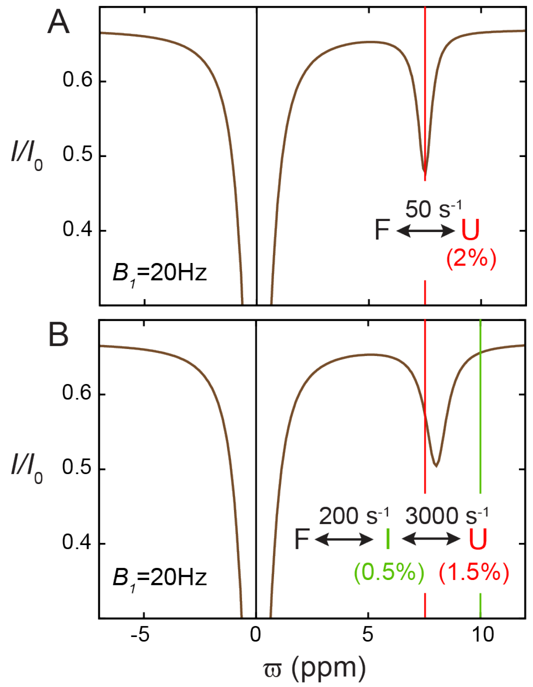

Fig. S1. Large changes in amide ^{15}N CEST profiles due to exchange between sparsely populated states on the intermediate exchange time-scale. (*A*) ^{15}N CEST profile calculated for the two-state F ⇋ U exchange process shown in the figure. There is a clear dip at ϖ_U (7.5 ppm; red line) even though p_U is only 2%. (*B*) ^{15}N CEST profile calculated for the three-state F ⇋ I ⇋ U exchange process in which the F to U interconversion proceeds via a sparsely populated intermediate I. The U state dip has broadened and shifted towards ϖ_I (10 ppm; green line); broadening of minor state dips is thus an indicator of minor exchange. Such CEST profiles can be analysed in a two-state (F ⇋ U) manner with the resulting best-fit parameters reporting on exchange between state F and a second state that is a composite of U and I. The best fit R_2 values of state U will be elevated (beyond what would be expected based on the size of the biomolecule), and consequently elevated minor state R_2 values are a sign of minor exchange (4, 11, 24). The calculations were performed with ϖ_F = 0 ppm (black line), B_0 = 16.4 T, B_1 = 20 Hz, $R_{1,F} = R_{1,U} = R_{1,I}$ = 1 s^{-1}, $R_{2,F} = R_{2,U} = R_{2,I}$ = 10 s^{-1} and T_{EX} = 400 ms.

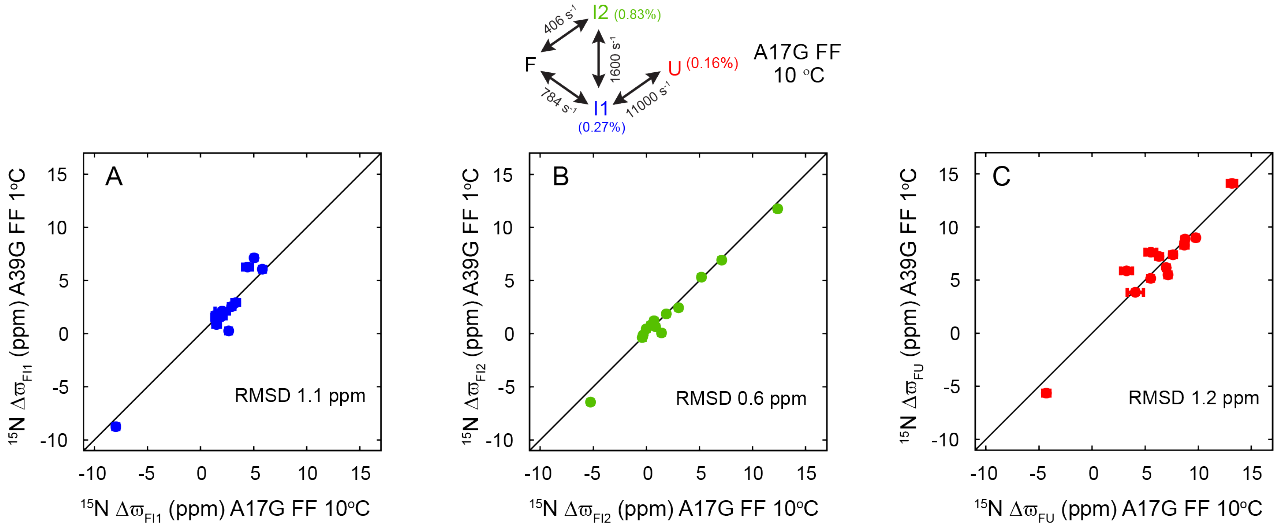

Fig. S2. Comparison of $\Delta\varpi_{FI1}$, $\Delta\varpi_{FI2}$, and $\Delta\varpi_{FU}$ values obtained from the four-state analysis of A17G FF amide ^{15}N CEST data recorded at 10 °C with the corresponding values for A39G FF obtained previously (1 °C) (11). For A17G FF the best-fit exchange parameters for the model shown on top are: $k_{ex,FI1}$ = 784 ± 67 s^{-1}, $k_{ex,FI2}$ = 406 ± 5 s^{-1}, $k_{ex,I1I2}$ = 1600 ± 113 s^{-1}, $k_{ex,I1U}$ = 11000 ± 1064 s^{-1}, p_{I1} = 0.27 ± 0.01%, p_{I2} = 0.83 ± 0.01% and p_U = 0.16 ± 0.02%. The excellent correlation between the $\Delta\varpi$ values from A39G and A17G FF establishes that both variants sample the same I1, I2 and U states. Additional details can be found in Table S4.

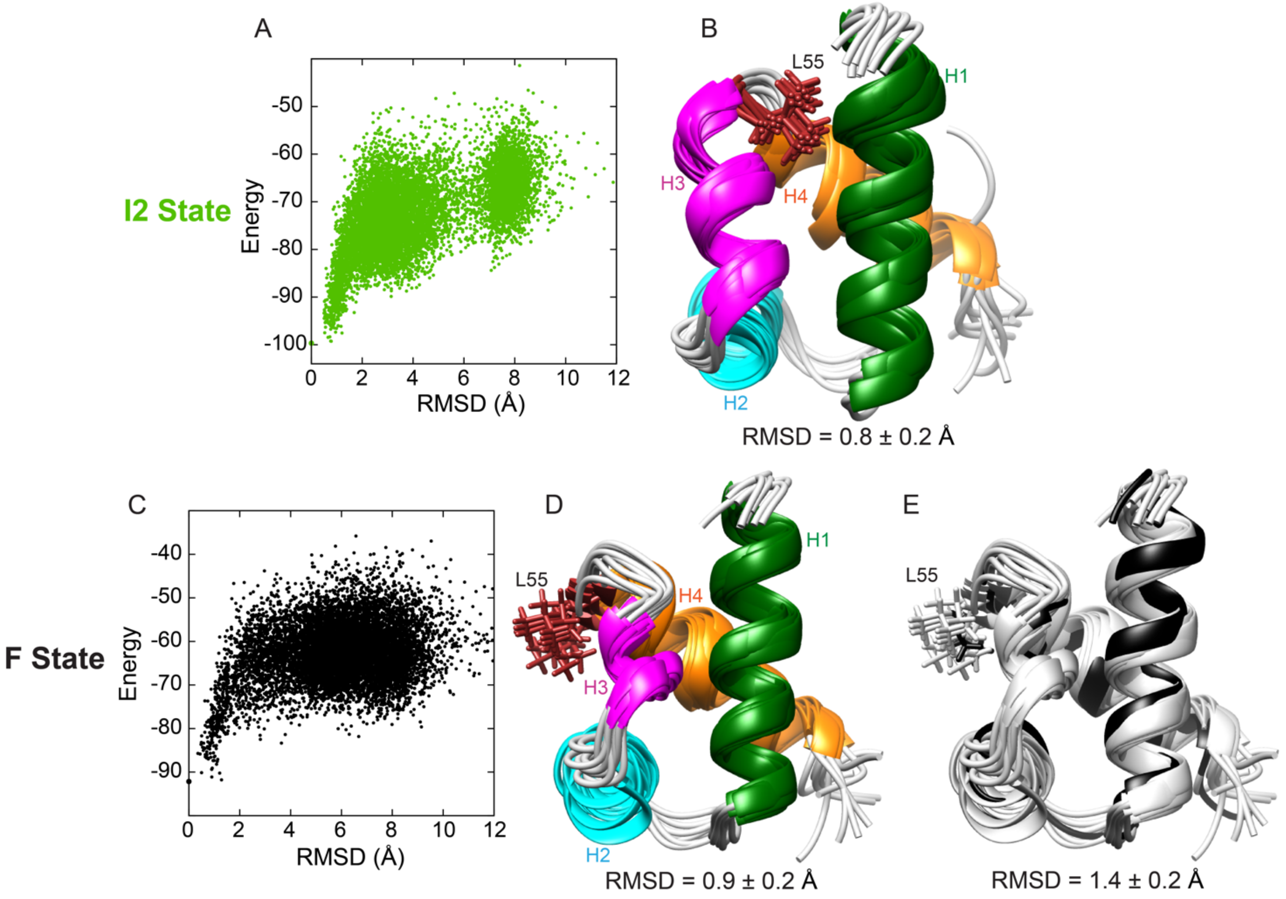

Fig. S3. Convergence of CS-ROSETTA structure calculations using CEST derived A17G FF I2 state chemical shifts. (*A*) Convergence plot of the CS-ROSETTA I2 state structure calculations. The rescaled CS-ROSETTA all-atom energy for each of the calculated structures is plotted against the C^{α} RMSD to the lowest energy structure. (*B*) The ten lowest energy models are tightly clustered (C^{α} RMSD < 2 Å) with a C^{α} RMSD to the lowest energy structure of 0.8 ± 0.2 Å, establishing convergence of the structure calculations. Note that the position of the sidechain of L55 is well defined. (*C-E*) Control calculations performed using the F state chemical shifts. The chemical shifts for a given atomic site was used in the F state CS-ROSETTA calculations only if the I2 state chemical shift for the same site was available. (*C*) Convergence plot of the CS-ROSETTA F state structure calculations. (*D*) The ten lowest energy models are tightly clustered with a C^{α} RMSD to the lowest energy structure is 0.9 ± 0.2 Å, showing that the F state structure calculations have also converged. The position of the sidechain of L55 is well defined and different from its position in (*B*). (*E*) Superposition of the ten lowest energy CS-ROSETTA A17G FF F state structures (grey) on the F state structure of WT FF (black; PDB: 1UZC) obtained using conventional NMR experiments (44); C^{α} RMSD: 1.4 ± 0.2 Å. The sidechain of L55 is in the same position in both the CS-ROSETTA derived A17G FF F state structural ensemble and the NOE-based WT FF structure. Only residues 11 to 68 that have RCI $S^2 \geq 0.65$ were used for the C^{α} RMSD calculations.

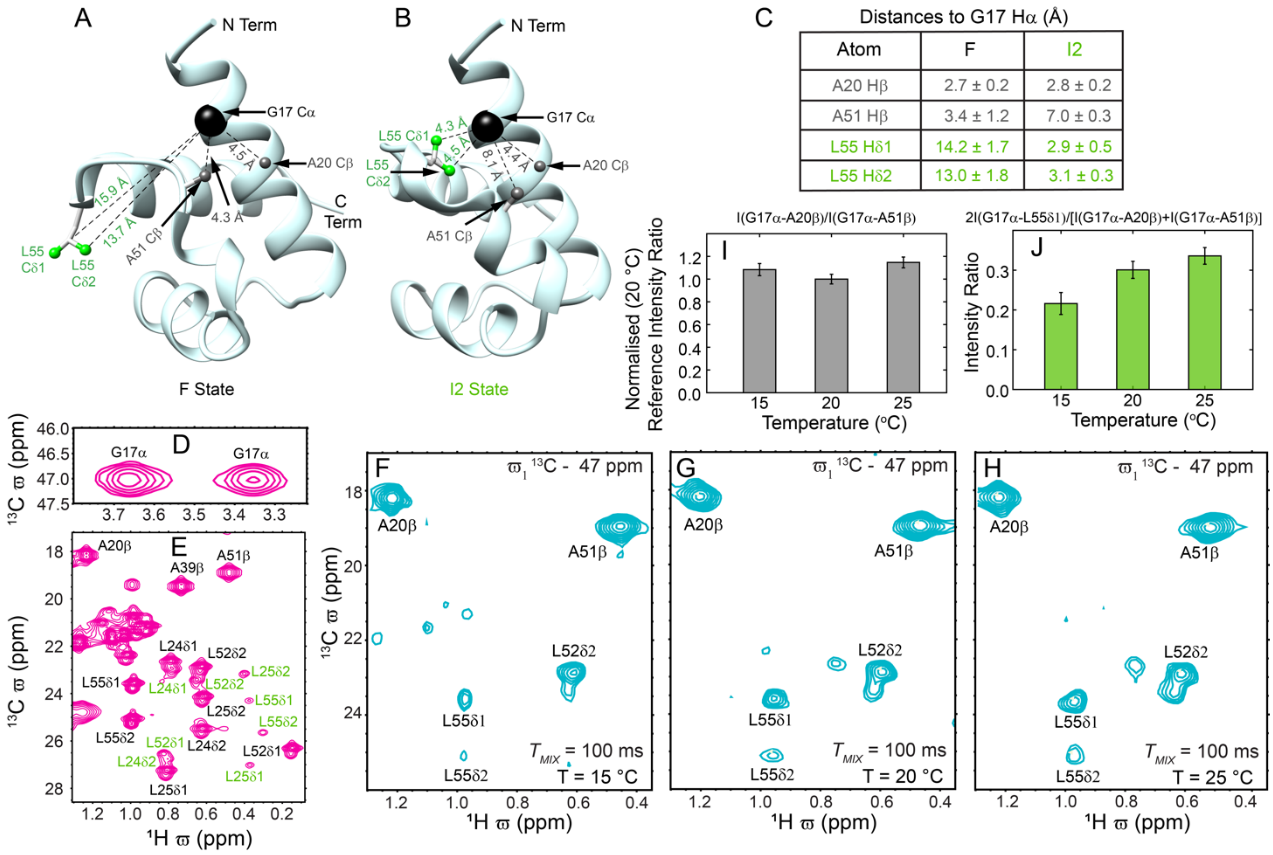

Fig. S4. Validating the CEST derived I2 state structure. (*A*,*B*,*C*) Distances between G17 C^{α} and A20 C^{β}, A51 C^{β}, and L55 δ1/ δ2 methyl group(s) in the F (*A*) and I2 (*B*) states. Note that the distances between G17 C^{α} and the L55 methyl δ groups that are large in F (~15 Å) become considerably smaller in I2 (~4.5 Å). The distances between G17 C^{α} and A20/A51 C^{β} are short in F (~4.5 Å) (*A*); these are used as references. The distance between G17 C^{α} and A51 C^{β} increases in I2 (~8.1 Å) (*B*). Distances are summarised in (*C*) where the mean and the standard deviation based on the ten calculated lowest energy structures is reported while in (*A*) and (*B*) distances from the lowest energy structure are shown. The Gly H^{α}-C^{α} (*D*) and methyl (*E*) regions of the ^{1}H-^{13}C correlation map of A17G S56P FF (20 °C). In (*E*) correlations arising from the I2 state are indicated in green. (*F-H*) Methyl region (ϖ_2, ϖ_3) extracted from a 3D HSQC(ϖ_1)-NOESY-HSQC (ϖ_2, ϖ_3) spectrum at ϖ_1 = 47 ppm, corresponding to the G17 $^{13}C^{\alpha}$ chemical shift. 3D NOESY datasets were recorded at 15 (*F*; $k_{ex,FI2}$ ~18.8 ± 0.9 s^{-1};p_{I2} ~20.4 ± 0.5 %), 20 (*G*; $k_{ex,FI2}$ ~35.1 ± 0.9 s^{-1};p_{I2} ~25.1 ± 0.4 %) and 25 (*H*; $k_{ex,FI2}$ ~62.8 ± 2 s^{-1};p_{I2} ~27.6 ± 0.4 %) °C. Correlations are only seen in the NOESY spectra for methyl protons proximal to the G17 H^{α} sites. (*I*) The intensity ratios of the G17H^{α}-A20H^{β} and G17H^{α}-A51H^{β} 'reference' NOEs do not change with temperature as these NOEs largely arises from the F state (short distances in F, long distance for G17H^{α}-A51H^{β} in I2, (*A*,*B*,*C*)). The intensity ratios at each of these three temperatures are scaled by the intensity ratio at 20 °C. (*J*) The intensity ratios of G17H^{α}-L55$H^{\delta 1}$ and the average of the G17H^{α}-A20H^{β}-and G17H^{α}-A51H^{β} correlations increase with temperature even as the rotational correlation time decreases, consistent with the G17-L55 NOEs arising from magnetization transfer in the I2 state as p_{I2} increases with temperature. The A17G S56P FF sample used in all these experiments is [U-^{15}N, ^{13}C] enriched everywhere except for the side-chains of Ile, Leu and Val that are [Ileδ1 - $^{13}CH_3$, ^{2}H; Leu, Val - $^{13}CH_3$/$^{12}CD_3$, ^{2}H] (sample 13). Hence methyl residues from sites other than Ileδ1, Leuδ1,δ2, Val γ1,γ2 are split in the methyl ^{13}C dimension of the ^{1}H-^{13}C HSQC correlation map that was recorded without a constant time period (*E*). Residual protonation at the carbon adjacent to the methyl group leads to a small shoulder in the ^{13}C dimension of the Ileδ1, Leuδ1,δ2, Val γ1,γ2 correlations.

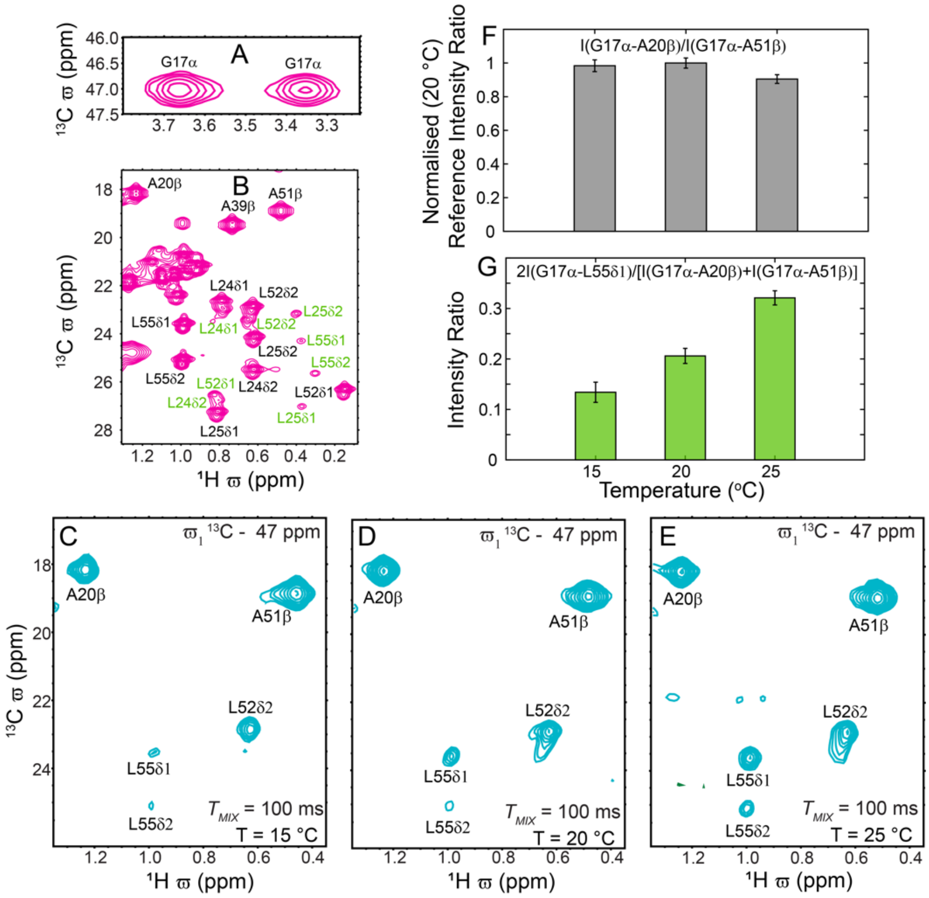

Fig S5. (*A*-*G*) are similar to Fig S4 (*D*-*J*), except that the protein (sample 14) was dissolved in 100% D_2O, 30% glucose buffer rather than 10% D_2O, 30% glucose buffer and a 3D HSQC-NOESY-HMQC rather than a 3D HSQC-NOESY-HSQC experiment was used to detect NOE correlations between G17 H^{α} and L55 H^{δ}. Two-state exchange parameters ($k_{ex,FI2}$, p_{I2}) obtained from methyl ^{13}C D-CEST experiments (17, 27) at 15, 20 and 25 °C are (20 ± 0.7 s^{-1}; 12.5 ± 0.2 %), (44.9 ± 1.5 s^{-1}; 16.4 ± 0.3 %) and (66 ± 3 s^{-1}; 20.9 ± 0.2 %) respectively.

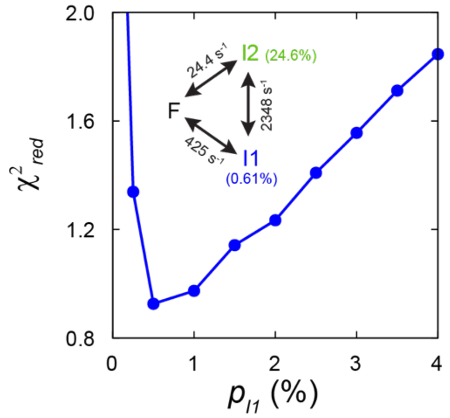

Fig. S6. χ^2_{red} vs p_{I1} obtained from a three-state analysis of A17G S56P FF amide ^{15}N CEST data recorded at 20 ^{o}C (B_1 = 3.2, 53.6, 107.3 and 214.5 Hz; 16.4 T; sample 13). A triangular three-state model was fit (χ^2_{red}~ 0.92, $k_{ex,FI1}$ = 425 ± 105 s^{-1}, $k_{ex,FI2}$ = 24.4 ± 4.1 s^{-1}, $k_{ex,I1I2}$ = 2348 ± 210 s^{-1}, p_{I1} = 0.61 ± 0.05 % and p_{I2} = 24.6 ± 1 %) to amide ^{15}N CEST data from I43, S50, L52, A53, K59 & V67. ^{15}N CEST experiments were carried out using the same sample (sample 13) that was used to record the NOESY experiments shown in Fig. 4F,G and *SI Appendix*, Fig. S4. In the calculations of the χ^2_{red} vs p_{I1} profile $\Delta\varpi_{FI1}$ values were held to within ±2 ppm of those determined previously from the four-state analysis of A39G FF (11).

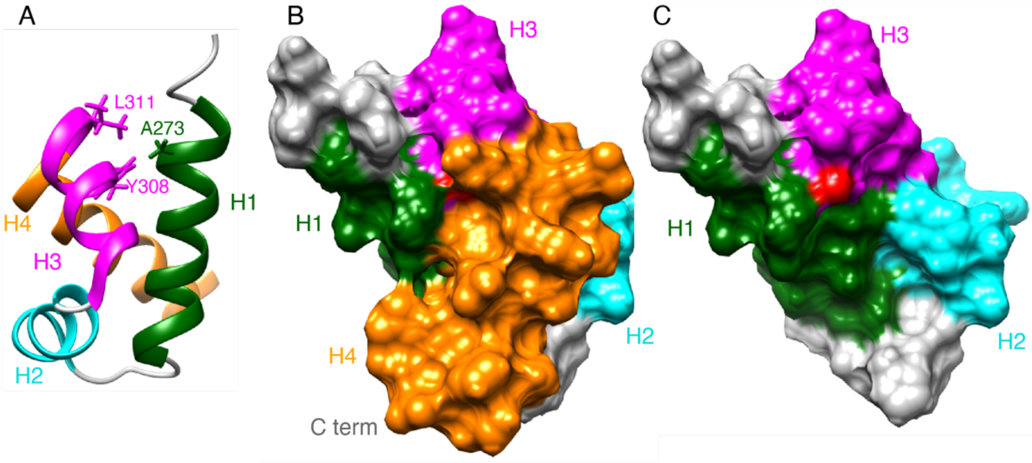

Fig. S7. (*A*) Ribbon representation of the native state of RhoGAPFF1 (PDB: 2k85) (47) illustrating the elongated H3 helix and that L311 from H3 is close to A273 from H1 in this construct akin to the proximity of L55 and A17 in the WT FF structure. Surface representation of RhoGAPFF1 in its native (*B*) and 'RhoGAPFF1 I1' states (*C*). In the native conformation the oxygen from the OH group of Y308 (coloured red) is inaccessible for phosphorylation (*B*). However when H4 is deleted, 'RhoGAPFF1 I1' state, (the structural equivalent of I1 where H4 is disordered), the oxygen from the OH group of Y308 (red) is clearly visible and accessible for modification (*C*). The molecular orientation in (*A*) differs from that in (*B*) and (*C*). In the HYPA/FBP11 FF domain, the equivalents of RhoGAPFF1 A273, Y308, and L311 are A17, L52, and L55, respectively.

Sample No	Mutant	Protein Labelling	Concentration (mM)	Buffer	Comments
**1**	A17G FF	[U-^{15}N, ^{13}C]	~4	50mM Sodium acetate, 100mM NaCl, 2mM EDTA, 2mM NaN_3, 20% [^{2}H]-glucose, 7% D_2O, pH 5.7	Used to obtain the ^{15}N, $^{13}C^{\alpha}$, $^{13}C^{O}$, $^{13}C^{\beta}$, $^1H^N$, $^1H^{\alpha}$ and methyl ^{13}C I2 state shifts.
**2**	A17G FF	[U-^{15}N, ^{13}C]	~4	50mM Sodium acetate,100mM NaCl, 2mM EDTA,2mM NaN_3, 20% [^{2}H]-glucose,100% D_2O, pH 5.7	Used to obtain the methyl ^{1}H, Gly $^{13}C^{\alpha}$, Ser/Thr $^{13}C^{\beta}$ shifts and also the $^{13}C^{\alpha}$/$^1H^{\alpha}$ shifts that were missing from sample 1.
**3**	A17G FF	[U-^{15}N], $^{13}C^{\alpha}$	~4	50mM Sodium acetate,100mM NaCl, 2mM EDTA, 2mM NaN_3, 20% [^{2}H]-glucose, 100% D_2O, pH 5.7	Used to obtain I2 state shift $^1H^{\alpha}$ and $^{13}C^{\alpha}$ of S56.
**4**	A17G FF	[U-^{15}N, ^{13}C], ~50% ^{2}H	~4	50mM Sodium acetate, 100mM NaCl, 2mM EDTA, 2mM NaN_3, 20% [^{2}H]-glucose, 100% D_2O, pH 5.7	Used to obtain I2 state shifts of G17 $^1H^{\alpha 1}$ and $^1H^{\alpha 2}$.
**5**	A17G FF	[U-^{15}N, ^{13}C], Ileδ_1-[$^{13}CH_3$], Leu, Val-[$^{13}CH_3$,$^{12}CD_3$]	~4	50mM Sodium acetate, 100mM NaCl, 2mM EDTA, 2mM NaN_3, 30% [^{2}H]-glucose, 10% D_2O, pH 5.7	Used to look for G17 $^1H^{\alpha}$-L55 $^1H^{\delta}$ NOEs.
**6**	A17G FF	[U-^{15}N]	~2	50mM Sodium acetate,100mM NaCl,2mM EDTA, 2mM NaN_3, 0M Urea, 10% D_2O, pH 5.7	Used for *m*-value analysis (2.5 ^{o}C) and to obtain four-state exchange parameters at 10 ^{o}C.
**7**	A17G FF	[U-^{15}N]	~2	50mM Sodium acetate, 100mM NaCl, 2mM EDTA, 2mM NaN_3, 0.25M Urea, 10% D_2O, pH 5.7	Used for *m*-value analysis (2.5 ^{o}C).
**8**	A17G FF	[U-^{15}N]	~2	50mM Sodium acetate, 100mM NaCl, 2mM EDTA, 2mM NaN_3, 0.5M Urea, 10% D_2O, pH 5.7	Used for *m*-value analysis (2.5 ^{o}C).
**9**	A17G FF	[U-^{15}N]	~2	50mM Sodium acetate, 100mM NaCl, 2mM EDTA, 2mM NaN_3, 0.75M Urea, 10% D_2O, pH 5.7	Used for *m*-value analysis (2.5 ^{o}C).
**10**	A17G FF	[U-^{15}N]	~2	50mM Sodium acetate, 100mM NaCl, 2mM EDTA, 2mM NaN_3, 1M urea, 10% D_2O , pH 5.7	Used for *m*-value analysis (2.5 ^{o}C).
**11**	A17G FF	[U-^{15}N]	~2	50mM Sodium acetate, 100mM NaCl, 2mM EDTA, 2mM NaN_3, 20% glucose, 10% D2O, pH 5.7	Used to show that the addition of glucose reduces the four-state A17G FF to a two-state system.
**12**	A17G S56P FF	[U-^{15}N]	~2	50mM Sodium acetate, 100mM NaCl, 2mM EDTA, 2mM NaN_3, 30% glucose, 10% D_2O, pH 5.7	Used to test if the S56P mutation has increased p_{I2}.
**13**	A17G S56P FF	[U-^{15}N, ^{13}C], Ileδ_1-[$^{13}CH_3$], Leu, Val-[$^{13}CH_3$,$^{12}CD_3$]	~4	50mM Sodium acetate, 100mM NaCl, 2mM EDTA, 2mM NaN_3, 30% [^{2}H] glucose, 10% D_2O, pH 5.7	Used to look for G17 H^{α}-L55 H^{δ} NOEs.
**14**	A17G S56P FF	[U-^{15}N, ^{13}C], Ileδ_1-[$^{13}CH_3$], Leu, Val-[$^{13}CH_3$,$^{12}CD_3$]	~4	50mM Sodium acetate, 100mM NaCl, 2mM EDTA, 2mM NaN_3, 30% [^{2}H] glucose, 100% D_2O, pH 5.7	Used to look for G17 H^{α}-L55 H^{δ} NOEs.

Table S1. Summary of the various A17G FF and A17G S56P FF NMR samples used in this study.

Sample No	Sample Details	CEST Experiment	B_1 (Hz)	T_{EX} (ms)	k_{ex} (s^{-1})	p_{I2} (%)	Comments
1	[U-^{15}N, ^{13}C] A17G FF<br><br>50mM Sodium acetate, 100mM NaCl, 2mM EDTA, 2mM NaN_3, 20% [^{2}H]-glucose, 7% D_2O, pH 5.7	^{15}N	31.7	350	630 ± 6	2.08 ± 0.01	$^1H^N$-^{15}N plane
			52.9	350			
		$^{13}C^{\alpha}$	40.9	350	Exchange parameters were fixed to the above values.		$^1H^N$-^{15}N plane
		$^{13}C^{O}$	46	350			$^1H^N$-^{15}N plane
		$^{13}C^{O}$	46	350			$^1H^N$-$^{13}C^O$ plane
		$^{13}C^{\beta}$	46	300			$^1H^N$-^{15}N plane
		$^1H^N$	35.8	350			$^1H^N$-^{15}N plane
		$^1H^{\alpha}$	46	350			$^1H^N$-^{15}N plane
		Methyl ^{13}C	45.3	350			^{1}H-^{13}C plane
2	[U-^{15}N, ^{13}C] A17G FF<br><br>50mM Sodium acetate,100mM NaCl,2mM EDTA,2mM NaN_3, 20% [^{2}H]-glucose, 100% D_2O, pH 5.7	$^{13}C^{\alpha}$	35.7	350	474 ± 21	1.71 ± 0.02	Used to obtain missing I2 state C^{α} shifts ($^1H^{\alpha}$-$^{13}C^{\alpha}$ plane).
			46	350			
		Gly $^{13}C^{\alpha}$	42.4	400	Exchange parameters were fixed to the above values.		$^1H^{\alpha}$-$^{13}C^{\alpha}$ plane
		Ser $^{13}C^{\beta}$	40.8	350			$^1H^{\beta}$-$^{13}C^{\beta}$ plane
		Thr $^{13}C^{\beta}$	46	350			$^1H^{\beta}$-$^{13}C^{\beta}$ plane
		$^1H^{\alpha}$	46	350			$^1H^{\alpha}$-$^{13}C^{\alpha}$ plane
		Methyl ^{1}H	46	400			^{1}H-^{13}C plane
3	[U-^{15}N], $^{13}C^{\alpha}$<br><br>50mM Sodium acetate,100mM NaCl,2mM EDTA,2mM NaN_3, 20% [^{2}H]-glucose, 100% D_2O, pH 5.7	$^{13}C^{\alpha}$	38.3	350	501 ± 28	1.67 ± 0.02	Obtain the S56 $^{13}C^{\alpha}$ shift ($^1H^{\alpha}$-$^{13}C^{\alpha}$ plane).
			49.3	350			
		$^1H^{\alpha}$	35.5	350	Exchange parameters were fixed to the above values.		Obtain the S56 $^1H^{\alpha}$ shift ($^1H^{\alpha}$-$^{13}C^{\alpha}$ plane).
			45.6	350			
4	[U-^{15}N, ^{13}C], ~50% ^{2}H<br><br>50mM Sodium acetate,100mM NaCl,2mM EDTA,2mM NaN_3, 20% [^{2}H]-glucose, 100% D_2O, pH 5.7	Gly $^1H^{\alpha}$	24.7	350	456 ± 70	1.85 ± 0.1	Gly $^1H^{\alpha}$ CEST experiment ($^1H^{\alpha}$-$^{13}C^{\alpha}$ plane).
			39.6	350			

Table S2. Summary of the (16.4 T) CEST NMR experiments carried out at 20 ^{o}C using various A17G FF samples to obtain the chemical shifts of the I2 state. Sample numbers are from Table S1.

Table S3. ϖ_F and the CEST derived $\Delta\varpi_{FI2}$ values of A17G FF. The I2 state chemical shifts (ϖ_{I2}) can be calculated from the F state chemical shifts (ϖ_F) and $\Delta\varpi_{FI2}$ values as $\varpi_{I2} = \varpi_F + \Delta\varpi_{FI2}$. Experiments were performed at 20 °C using the samples and experiments listed in tables S1 and S2 respectively.

Residue	Nucleus	ϖ_F (ppm)	$\Delta\varpi_{FI2}$ (ppm)
W11	C	175.27	0.6 ± 0.1
W11	Cα	56.98	-0.7 ± 0.1
W11	Cβ	29.29	0.0 ± 0.1
W11	Hα	4.802	0.00 ± 0.02
N12	C	175.35	0.3 ± 0.2
N12	Cα	54.65	-0.5 ± 0.1
N12	Cβ	40.27	0.0 ± 0.1
N12	HN	9.065	-0.15 ± 0.08
N12	Hα	4.762	0.13 ± 0.03
N12	N	120.05	-1.7 ± 0.1
T13	C	175.36	0.0 ± 0.1
T13	Cα	59.80	-0.4 ± 0.3
T13	Cβ	72.26	0.6 ± 0.2
T13	Cγ2	21.87	0.0 ± 0.1
T13	HN	7.774	0.08 ± 0.07
T13	Hα	4.733	-0.15 ± 0.03
T13	Hγ2	1.322	0.00 ± 0.01
T13	N	108.87	1.5 ± 0.2
K14	C	178.81	-0.3 ± 0.2
K14	Cα	58.74	0.8 ± 0.1
K14	Cβ	31.58	0.0 ± 0.2
K14	HN	8.957	-0.18 ± 0.09
K14	Hα	4.240	0.14 ± 0.03
K14	N	123.13	-0.8 ± 0.1
E15	C	179.22	0.4 ± 0.3
E15	Cα	59.61	0.0 ± 0.2
E15	Cβ	28.99	0.0 ± 0.1
E15	HN	8.533	-0.15 ± 0.06
E15	Hα	4.019	0.19 ± 0.01
E15	N	119.23	0.2 ± 0.1
E16	C	179.99	0.3 ± 0.2
E16	Cα	59.08	0.0 ± 0.1
E16	Cβ	30.54	0.0 ± 0.1
E16	HN	8.001	-0.15 ± 0.05
E16	Hα	4.163	-0.20 ± 0.02
E16	N	119.82	-0.7 ± 0.1
G17	C	174.77	0.0 ± 0.1
G17	Cα	47.09	-0.2 ± 0.2
G17	HN	8.369	-0.13 ± 0.02
G17	Hα2	3.349	-0.46 ± 0.01
G17	Hα1	3.660	-0.90 ± 0.01
G17	N	109.75	-0.3 ± 0.1
K18	C	178.87	0.6 ± 0.3
K18	Cα	60.29	0.0 ± 0.1
K18	Cβ	32.50	0.0 ± 0.1
K18	HN	8.346	-0.13 ± 0.01
K18	Hα	3.761	0.22 ± 0.01
K18	N	121.31	0.0 ± 0.1
Q19	C	178.06	0.0 ± 0.1
Q19	Cα	58.67	0.1 ± 0.1
Q19	Cβ	27.89	0.0 ± 0.1
Q19	HN	7.842	-0.15 ± 0.01
Q19	Hα	4.043	0.23 ± 0.01

Q19	N	118.41	0.2 ± 0.1
A20	C	178.95	0.8 ± 0.2
A20	Cα	54.89	0.0 ± 0.1
A20	Cβ	18.18	0.0 ± 0.1
A20	HN	7.947	-0.19 ± 0.12
A20	Hα	4.124	-0.16 ± 0.05
A20	Hβ	1.286	0.08 ± 0.02
A20	N	122.53	-0.3 ± 0.1
F21	C	177.08	0.4 ± 0.1
F21	Cα	61.79	0.7 ± 0.2
F21	Cβ	40.36	0.0 ± 0.2
F21	HN	8.106	0.35 ± 0.07
F21	Hα	3.746	0.00 ± 0.02
F21	N	119.06	0.5 ± 0.1
K22	C	178.90	0.0 ± 0.1
K22	Cα	60.45	0.0 ± 0.2
K22	Cβ	32.00	0.0 ± 0.1
K22	HN	8.019	-0.16 ± 0.01
K22	Hα	3.886	0.20 ± 0.01
K22	N	117.43	0.4 ± 0.1
E23	C	178.52	0.2 ± 0.2
E23	Cα	59.35	0.0 ± 0.1
E23	Cβ	29.15	0.0 ± 0.1
E23	HN	8.284	-0.18 ± 0.09
E23	Hα	3.916	0.00 ± 0.03
E23	N	120.20	-0.9 ± 0.1
L24	C	176.64	1.2 ± 0.1
L24	Cα	58.24	0.5 ± 0.1
L24	Cβ	40.95	0.0 ± 0.1
L24	Cδ1	22.93	-0.4 ± 0.2
L24	Cδ2	25.85	-0.2 ± 0.3
L24	HN	7.746	-0.16 ± 0.05
L24	Hα	3.753	0.00 ± 0.02
L24	Hδ1	0.882	0.12 ± 0.02
L24	Hδ2	0.713	0.11 ± 0.03
L24	N	122.78	-0.3 ± 0.1
L25	C	177.64	0.4 ± 0.1
L25	Cα	57.97	0.0 ± 0.1
L25	Cβ	40.80	0.0 ± 0.1
L25	Cδ1	27.25	0.0 ± 0.2
L25	Cδ2	24.24	-0.9 ± 0.1
L25	HN	7.211	-0.15 ± 0.08
L25	Hα	3.350	0.15 ± 0.02
L25	Hδ1	0.868	-0.41 ± 0.02
L25	Hδ2	0.675	0.00 ± 0.04
L25	N	117.59	0.2 ± 0.1
K26	C	181.22	0.0 ± 0.2
K26	Cα	58.97	-0.3 ± 0.2
K26	Cβ	33.05	0.0 ± 0.1
K26	HN	7.543	-0.04 ± 0.05
K26	Hα	4.239	0.21 ± 0.01
K26	N	115.56	2.2 ± 0.1
E27	C	179.10	0.0 ± 0.2
E27	Cα	59.47	0.0 ± 0.1
E27	Cβ	30.35	0.3 ± 0.2
E27	HN	9.017	-0.34 ± 0.01
E27	Hα	3.978	0.33 ± 0.01
E27	N	122.66	-0.1 ± 0.1
K28	C	174.54	0.0 ± 0.1
K28	Cα	54.12	0.0 ± 0.2
K28	Cβ	30.70	0.0 ± 0.1

K28	HN	7.760	-0.15 ± 0.08
K28	Hα	4.220	0.21 ± 0.01
K28	N	114.05	1.2 ± 0.1
R29	C	176.50	0.4 ± 0.1
R29	Cα	55.87	-0.2 ± 0.1
R29	Cβ	26.66	0.0 ± 0.1
R29	HN	7.914	-0.17 ± 0.04
R29	Hα	3.867	0.22 ± 0.05
R29	N	115.37	-0.1 ± 0.1
V30	Cα	61.68	-0.3 ± 0.2
V30	Cγ1	21.19	-0.5 ± 0.1
V30	Cγ2	22.48	0.0 ± 0.1
V30	HN	7.567	-0.10 ± 0.06
V30	Hα	3.897	-0.43 ± 0.05
V30	Hγ1	1.220	-0.09 ± 0.02
V30	Hγ2	1.107	0.12 ± 0.02
V30	N	120.30	-0.4 ± 0.1
P31	C	177.53	0.0 ± 0.1
P31	Cα	63.38	-0.3 ± 0.1
P31	Cβ	32.86	-0.4 ± 0.2
P31	Hα	4.635	0.22 ± 0.01
S32	C	173.89	0.0 ± 0.1
S32	Cα	60.86	0.4 ± 0.3
S32	Cβ	62.68	0.8 ± 0.3
S32	HN	8.777	-0.16 ± 0.04
S32	Hα	3.998	0.24 ± 0.01
S32	N	115.90	0.2 ± 0.1
N33	C	175.81	0.0 ± 0.1
N33	Cα	51.61	-0.3 ± 0.1
N33	Cβ	37.78	0.0 ± 0.1
N33	HN	7.832	-0.18 ± 0.09
N33	Hα	4.819	0.17 ± 0.02
N33	N	115.24	-0.2 ± 0.1
A34	C	177.66	0.0 ± 0.1
A34	Cα	52.42	0.5 ± 0.2
A34	Cβ	19.59	0.0 ± 0.1
A34	HN	7.534	-0.15 ± 0.01
A34	Hα	4.464	0.25 ± 0.02
A34	Hβ	1.610	-0.02 ± 0.01
A34	N	122.75	-0.2 ± 0.2
S35	C	175.47	0.4 ± 0.1
S35	Cα	56.69	0.0 ± 0.1
S35	Cβ	65.45	0.0 ± 0.4
S35	HN	8.322	-0.16 ± 0.07
S35	Hα	4.884	0.00 ± 0.08
S35	N	115.79	0.0 ± 0.1
W36	C	175.92	0.6 ± 0.1
W36	Cα	59.63	0.0 ± 0.1
W36	HN	9.379	-0.17 ± 0.03
W36	Hα	3.980	0.15 ± 0.01
W36	N	122.56	0.8 ± 0.1
E37	C	178.96	0.4 ± 0.1
E37	Cα	60.89	-0.7 ± 0.1
E37	Cβ	28.63	0.0 ± 0.1
E37	HN	8.621	-0.22 ± 0.08
E37	Hα	3.329	0.22 ± 0.01
E37	N	115.97	-0.2 ± 0.1
Q38	C	178.32	0.0 ± 0.1
Q38	Cα	58.17	0.0 ± 0.1
Q38	Cβ	28.67	0.0 ± 0.1
Q38	HN	7.440	-0.14 ± 0.08

Q38	Hα	3.835	0.20 ± 0.03
Q38	N	117.71	1.8 ± 0.1
A39	C	179.28	0.0 ± 0.3
A39	Cα	54.96	0.0 ± 0.1
A39	Cβ	19.47	0.0 ± 0.1
A39	HN	8.117	-0.14 ± 0.08
A39	Hα	3.652	0.17 ± 0.02
A39	Hβ	0.817	0.00 ± 0.02
A39	N	122.17	-0.2 ± 0.1
M40	C	177.28	1.3 ± 0.1
M40	Cα	59.15	-2.7 ± 0.1
M40	Cβ	31.43	0.0 ± 0.3
M40	Cε	16.99	-0.6 ± 0.1
M40	HN	8.397	-0.45 ± 0.01
M40	Hα	3.040	0.74 ± 0.01
M40	Hε	2.010	-0.50 ± 0.01
M40	N	116.35	-1.9 ± 0.1
K41	C	178.26	0.3 ± 0.1
K41	Cα	58.85	-0.3 ± 0.1
K41	Cβ	32.38	0.0 ± 0.1
K41	HN	6.764	0.47 ± 0.01
K41	Hα	3.702	0.23 ± 0.01
K41	N	114.10	3.2 ± 0.1
M42	C	178.00	0.3 ± 0.2
M42	Cα	57.95	0.0 ± 0.2
M42	Cβ	34.31	-1.9 ± 0.4
M42	Cε	17.34	0.0 ± 0.1
M42	HN	7.322	0.14 ± 0.09
M42	Hα	4.132	0.00 ± 0.02
M42	Hε	2.159	0.00 ± 0.04
M42	N	113.94	0.8 ± 0.1
I43	C	175.84	0.9 ± 0.1
I43	Cα	62.11	0.0 ± 0.1
I43	Cβ	39.04	0.0 ± 0.2
I43	Cγ2	16.87	0.0 ± 0.1
I43	Cδ	13.86	0.4 ± 0.1
I43	HN	7.101	0.30 ± 0.02
I43	Hα	4.350	0.04 ± 0.07
I43	Hγ2	0.544	-0.07 ± 0.02
I43	Hδ	0.696	0.00 ± 0.03
I43	N	108.94	1.7 ± 0.1
I44	C	174.51	0.6 ± 0.1
I44	Cα	63.12	-0.4 ± 0.1
I44	Cβ	38.02	0.0 ± 0.1
I44	Cγ2	15.73	0.9 ± 0.1
I44	Cδ	14.69	0.3 ± 0.1
I44	HN	7.197	-0.06 ± 0.01
I44	Hα	2.851	0.36 ± 0.02
I44	Hγ2	0.635	0.00 ± 0.03
I44	Hδ	0.624	0.00 ± 0.11
I44	N	119.29	0.0 ± 0.1
N45	C	174.61	0.5 ± 0.1
N45	Cα	52.96	-0.1 ± 0.1
N45	Cβ	38.27	0.0 ± 0.1
N45	HN	7.771	-0.23 ± 0.09
N45	Hα	4.708	0.30 ± 0.01
N45	N	116.29	0.5 ± 0.1
D46	Cα	51.72	0.6 ± 0.2
D46	HN	7.677	0.31 ± 0.01
D46	Hα	4.976	0.10 ± 0.03
D46	N	123.25	1.6 ± 0.1

P47	C	178.80	-0.4 ± 0.1
P47	Cα	65.41	-0.4 ± 0.1
P47	Cβ	32.69	0.0 ± 0.1
P47	Hα	4.515	0.22 ± 0.04
R48	C	177.22	0.8 ± 0.2
R48	Cα	57.87	-0.4 ± 0.3
R48	Cβ	30.20	-1.7 ± 0.4
R48	HN	9.778	-0.30 ± 0.01
R48	Hα	3.962	0.42 ± 0.02
R48	N	115.95	1.6 ± 0.1
Y49	C	176.69	0.6 ± 0.1
Y49	Cα	61.37	-0.4 ± 0.2
Y49	Cβ	38.52	0.0 ± 0.1
Y49	HN	8.452	-0.17 ± 0.04
Y49	Hα	4.028	0.22 ± 0.01
Y49	N	121.70	-3.4 ± 0.1
S50	C	173.92	2.8 ± 0.1
S50	Cα	58.06	3.7 ± 0.1
S50	Cβ	63.39	-1.3 ± 0.1
S50	HN	7.291	0.50 ± 0.01
S50	Hα	4.326	-0.36 ± 0.04
S50	N	108.01	5.2 ± 0.1
A51	C	176.80	2.7 ± 0.1
A51	Cα	54.39	0.5 ± 0.1
A51	Cβ	19.02	0.0 ± 0.1
A51	HN	7.513	0.28 ± 0.01
A51	Hα	3.379	0.94 ± 0.01
A51	Hβ	0.565	1.09 ± 0.01
A51	N	123.39	-1.9 ± 0.1
L52	C	175.39	2.3 ± 0.1
L52	Cα	52.28	3.0 ± 0.1
L52	Cβ	43.25	0.0 ± 0.2
L52	Cδ1	26.13	0.6 ± 0.1
L52	Cδ2	23.11	-0.4 ± 0.1
L52	HN	5.924	1.77 ± 0.01
L52	Hα	4.343	0.58 ± 0.02
L52	Hδ1	0.213	0.78 ± 0.01
L52	Hδ2	0.699	-0.10 ± 0.03
L52	N	111.95	6.6 ± 0.1
A53	C	178.73	0.6 ± 0.1
A53	Cα	54.85	0.6 ± 0.1
A53	Cβ	20.07	-1.7 ± 0.1
A53	HN	8.582	-1.04 ± 0.01
A53	Hα	3.978	-0.23 ± 0.02
A53	Hβ	1.497	-0.12 ± 0.01
A53	N	125.61	-2.9 ± 0.1
K54	C	177.46	0.8 ± 0.2
K54	Cα	55.46	-0.7 ± 0.2
K54	Cβ	33.85	-1.7 ± 0.2
K54	HN	7.953	-0.16 ± 0.05
K54	Hα	4.432	-0.08 ± 0.07
K54	N	115.28	-1.0 ± 0.1
L55	C	178.14	0.8 ± 0.2
L55	Cα	58.49	-3.0 ± 0.1
L55	Cβ	41.03	1.4 ± 0.3
L55	Cδ1	23.57	-0.5 ± 0.2
L55	Cδ2	25.42	0.0 ± 0.1
L55	HN	8.979	-1.41 ± 0.01
L55	Hα	3.695	0.60 ± 0.01
L55	Hδ1	1.042	-0.44 ± 0.02
L55	Hδ2	1.027	-0.65 ± 0.01

L55	N	127.78	-5.6 ± 0.1
S56	C	177.34	-2.2 ± 0.1
S56	Cα	61.01	-1.2 ± 0.1
S56	HN	8.677	-0.19 ± 0.09
S56	Hα	3.801	0.61 ± 0.01
S56	N	112.05	12.0 ± 0.1
E57	C	179.17	-2.6 ± 0.1
E57	Cα	58.52	-1.9 ± 0.1
E57	Cβ	30.24	-2.3 ± 0.1
E57	HN	6.623	1.46 ± 0.01
E57	Hα	3.967	0.48 ± 0.02
E57	N	121.59	-3.6 ± 0.1
K58	C	177.96	0.0 ± 0.2
K58	Cα	59.38	0.9 ± 0.1
K58	Cβ	34.01	-1.8 ± 0.6
K58	HN	7.584	0.52 ± 0.02
K58	Hα	2.968	0.40 ± 0.02
K58	N	121.38	-2.5 ± 0.1
K59	C	179.03	0.5 ± 0.1
K59	Cα	60.09	0.0 ± 0.2
K59	Cβ	31.91	0.0 ± 0.1
K59	HN	7.835	0.48 ± 0.01
K59	Hα	3.447	0.35 ± 0.04
K59	N	116.45	3.1 ± 0.1
Q60	C	178.92	0.0 ± 0.2
Q60	Cα	59.13	0.0 ± 0.1
Q60	Cβ	28.41	-0.5 ± 0.2
Q60	HN	7.626	0.42 ± 0.01
Q60	Hα	4.098	0.46 ± 0.02
Q60	N	119.05	0.6 ± 0.1
A61	C	180.28	-1.5 ± 0.1
A61	Cα	54.94	0.5 ± 0.2
A61	Cβ	18.40	0.0 ± 0.1
A61	HN	7.853	-0.19 ± 0.11
A61	Hα	4.243	0.14 ± 0.01
A61	Hβ	1.514	0.12 ± 0.02
A61	N	123.09	-0.1 ± 0.1
F62	C	176.27	0.3 ± 0.2
F62	Cα	60.29	0.0 ± 0.2
F62	Cβ	39.45	0.0 ± 0.1
F62	HN	8.681	-0.17 ± 0.05
F62	Hα	4.640	0.18 ± 0.01
F62	N	120.13	-0.6 ± 0.1
N63	C	177.60	0.4 ± 0.3
N63	Cα	56.36	0.0 ± 0.1
N63	Cβ	37.76	0.0 ± 0.1
N63	HN	8.617	-0.19 ± 0.01
N63	Hα	4.103	-0.10 ± 0.03
N63	N	118.95	0.4 ± 0.1
A64	C	179.65	0.3 ± 0.2
A64	Cα	54.80	0.0 ± 0.1
A64	Cβ	18.17	0.0 ± 0.1
A64	Hα	4.145	-0.14 ± 0.05
A64	Hβ	1.564	0.01 ± 0.03
Y65	C	177.44	0.0 ± 0.2
Y65	Cα	60.34	0.0 ± 0.2
Y65	Cβ	38.91	0.0 ± 0.1
Y65	HN	8.080	-0.29 ± 0.09
Y65	Hα	4.239	0.16 ± 0.02
Y65	N	120.83	0.0 ± 0.2
K66	C	177.84	0.3 ± 0.2

K66	Cα	60.01	0.0 ± 0.1
K66	Cβ	32.04	0.0 ± 0.1
K66	HN	8.000	-0.20 ± 0.01
K66	Hα	3.492	0.21 ± 0.01
K66	N	119.09	-0.1 ± 0.1
V67	C	177.14	0.0 ± 0.1
V67	Cα	63.41	-0.2 ± 0.1
V67	Cβ	31.90	0.0 ± 0.1
V67	Cγ1	21.31	0.0 ± 0.1
V67	Cγ2	20.91	0.0 ± 0.1
V67	HN	7.249	-0.14 ± 0.01
V67	Hα	4.004	0.22 ± 0.03
V67	Hγ1	0.971	-0.07 ± 0.01
V67	Hγ2	1.043	0.00 ± 0.07
V67	N	114.19	1.3 ± 0.1
Q68	C	176.53	-0.4 ± 0.3
Q68	Cα	56.59	0.4 ± 0.1
Q68	Cβ	28.87	0.0 ± 0.1
Q68	HN	7.682	-0.14 ± 0.01
Q68	Hα	4.256	-0.13 ± 0.03
Q68	N	120.45	-0.5 ± 0.2
T69	C	174.62	0.2 ± 0.1
T69	Cα	62.53	0.3 ± 0.1
T69	Cβ	69.53	0.0 ± 0.1
T69	Cγ2	21.55	0.0 ± 0.1
T69	HN	7.847	-0.16 ± 0.08
T69	Hα	4.179	0.00 ± 0.04
T69	Hγ2	1.110	0.02 ± 0.02
T69	N	113.91	0.3 ± 0.1
E70	C	175.47	0.0 ± 0.1
E70	Cα	56.75	-0.2 ± 0.1
E70	Cβ	30.09	0.0 ± 0.1
E70	HN	8.075	-0.19 ± 0.06
E70	Hα	4.262	0.08 ± 0.03
E70	N	123.26	-0.4 ± 0.1
K71	Cα	57.58	0.2 ± 0.1
K71	HN	7.856	-0.14 ± 0.08
K71	Hα	4.128	0.11 ± 0.05
K71	N	127.07	0.1 ± 0.1

Sample No	Protein Labelling	Buffer	Temperature (°C)	Experimental Parameters		Fitted Parameters							Comments
				B_1 (Hz)	T_{EX} (ms)	$k_{ex,FI1}$ (s^{-1})	$k_{ex,FI2}$ (s^{-1})	$k_{ex,I1I2}$ (s^{-1})	$k_{ex,I1U}$ (s^{-1})	p_{I1} (%)	p_{I2} (%)	p_U (%)	
6	[U-^{15}N]	50mM Sodium acetate,100mM NaCl,2mM EDTA, 2mM NaN$_3$, 10% D$_2$O, pH 5.7	10	26	450	784 ± 67	406 ± 5	1600 ± 113	11000 ± 1064	0.27 ± 0.01	0.83 ± 0.01	0.16 ± 0.02	Data from residues T13, K26, K28, R29, N33, E37, K41, M42, I43, S50, L52, L55, and S56 was used.
				52.1	400								
				104.1	350								
				208.3	350								
6	[U-^{15}N]	50mM Sodium acetate,100mM NaCl,2mM EDTA, 2mM NaN$_3$, 10% D$_2$O, pH 5.7	2.5	26	450	372 ± 21	216 ± 3	886 ± 39	8370 ± 610	0.13 ± 0.01	0.55 ± 0.01	0.11 ± 0.01	For *m*-value analysis<br><br>Data from residues T13, K26, K28, R29, N33, E37, K41, M42, I43, S50, L52, L55, and S56 was used.
				51.7	400								
				103.5	350								
				207	350								
7	[U-^{15}N]	50mM Sodium acetate,100mM NaCl,2mM EDTA, 2mM NaN$_3$, 0.25 M urea, 10% D$_2$O, pH 5.7		26	450	385 ± 16	225 ± 2	836 ± 30	8890 ± 462	0.19 ± 0.01	0.52 ± 0.01	0.25 ± 0.01	
				51.9	400								
				103.7	350								
				207.4	350								
8	[U-^{15}N]	50mM Sodium acetate,100mM NaCl,2mM EDTA, 2mM NaN$_3$, 0.50 M urea, 10% D$_2$O, pH 5.7		26	450	371 ± 15	236 ± 3	828 ± 26	9490 ± 351	0.25 ± 0.01	0.52 ± 0.01	0.52 ± 0.01	
				52	400								
				104.1	350								
				208.2	350								
9	[U-^{15}N]	50mM Sodium acetate,100mM NaCl,2mM EDTA, 2mM NaN$_3$, 0.75 M urea, 10% D$_2$O, pH 5.7		26	450	345 ± 16	249 ± 3	853 ± 27	9510 ± 346	0.34 ± 0.01	0.51 ± 0.01	1.11 ± 0.02	
				51.9	400								
				103.8	350								
				207.5	350								
10	[U-^{15}N]	50mM Sodium acetate,100mM NaCl,2mM EDTA, 2mM NaN$_3$, 1.0 M Urea, 10% D$_2$O, pH 5.7		26	450	322 ± 18	256 ± 4	885 ± 30	9290 ± 370	0.43 ± 0.02	0.51 ± 0.01	2.06 ± 0.03	
				52.2	400								
				104.3	350								
				208.6	350								

Table S4. Four-state exchange parameters obtained by analysing A17G FF ^{15}N CEST data under different conditions. Sample numbers are from Table S1.

Legend for supplementary file A17GFF_I2_lowest10.pdb. This file contains the coordinates of the ten lowest energy A17G FF I2 state structures obtained using the CS-ROSETTA program (41) as described in the Materials and Methods section of the *SI Appendix*.

References

1. J. Cavanagh, W. J. Fairbrother, A. G. Palmer, M. Rance, N. J. Skelton, *Protein NMR Spectroscopy, Principles and Practice* (Academic Press, ed. 2nd 2006).

2. S. Forsen, R. A. Hoffman, Study of Moderately Rapid Chemical Exchange Reactions by Means of Nuclear Magnetic Double Resonance. *J Chem Phys* **39**, 2892-2901 (1963).

3. K. M. Ward, A. H. Aletras, R. S. Balaban, A new class of contrast agents for MRI based on proton chemical exchange dependent saturation transfer (CEST). *J Magn Reson* **143**, 79-87 (2000).

4. P. Vallurupalli, G. Bouvignies, L. E. Kay, Studying "invisible" excited protein States in slow exchange with a major state conformation. *J Am Chem Soc* **134**, 8148-8161 (2012).

5. P. Vallurupalli, A. Sekhar, T. Yuwen, L. E. Kay, Probing conformational dynamics in biomolecules via chemical exchange saturation transfer: a primer. *J Biomol NMR* **67**, 243-271 (2017).

6. N. P. Khandave, D. F. Hansen, P. Vallurupalli, Increasing the accuracy of exchange parameters reporting on slow dynamics by performing CEST experiments with 'high' B(1) fields. *J Magn Reson* **363**, 107699 (2024).

7. N. P. Khandave, A. Sekhar, P. Vallurupalli, Studying micro to millisecond protein dynamics using simple amide (15)N CEST experiments supplemented with major-state R(2) and visible peak-position constraints. *J Biomol NMR* 10.1007/s10858-023-00419-2 (2023).

8. A. Rangadurai, E. S. Szymaski, I. J. Kimsey, H. Shi, H. Al-Hashimi, Characterizing micro-to-millisecond chemical exchange in nucleic acids using off-resonance R1ρ relaxation dispersion. *Progress in Nuclear Magnetic Resonance Spectroscopy* **112-113**, 55-102 (2019).

9. T. Yuwen, L. E. Kay, G. Bouvignies, Dramatic Decrease in CEST Measurement Times Using Multi-Site Excitation. *Chemphyschem* **19**, 1707-1710 (2018).

10. V. P. Tiwari, D. De, N. Thapliyal, L. E. Kay, P. Vallurupalli, Beyond slow two-state protein conformational exchange using CEST: applications to three-state protein interconversion on the millisecond timescale. *J Biomol NMR* 10.1007/s10858-023-00431-6 (2024).

11. V. P. Tiwari, Y. Toyama, D. De, L. E. Kay, P. Vallurupalli, The A39G FF domain folds on a volcano-shaped free energy surface via separate pathways. *Proc Natl Acad Sci U S A* **118** (2021).

12. P. Lundstrom, P. Vallurupalli, D. F. Hansen, L. E. Kay, Isotope labeling methods for studies of excited protein states by relaxation dispersion NMR spectroscopy. *Nat Protoc* **4**, 1641-1648 (2009).

13. N. K. Goto, K. H. Gardner, G. A. Mueller, R. C. Willis, L. E. Kay, A robust and cost-effective method for the production of Val, Leu, Ile (delta 1) methyl-protonated 15N-, 13C-, 2H-labeled proteins. *J Biomol NMR* **13**, 369-374 (1999).

14. D. F. Hansen, P. Vallurupalli, P. Lundstrom, P. Neudecker, L. E. Kay, Probing chemical shifts of invisible states of proteins with relaxation dispersion NMR spectroscopy: how well can we do? *J Am Chem Soc* **130**, 2667-2675 (2008).

15. V. P. Tiwari, P. Vallurupalli, A CEST NMR experiment to obtain glycine (1)H(alpha) chemical shifts in 'invisible' minor states of proteins. *J Biomol NMR* **74**, 443-455 (2020).

16. P. Vallurupalli, D. F. Hansen, P. Lundstrom, L. E. Kay, CPMG relaxation dispersion NMR experiments measuring glycine 1H alpha and 13C alpha chemical shifts in the 'invisible' excited states of proteins. *J Biomol NMR* **45**, 45-55 (2009).

17. G. Bouvignies, P. Vallurupalli, L. E. Kay, Visualizing Side Chains of Invisible Protein Conformers by Solution NMR. *Journal of Molecular Biology* **426**, 763-774 (2014).

18. P. Vallurupalli, G. Bouvignies, L. E. Kay, A Computational Study of the Effects of C-13-C-13 Scalar Couplings on C-13 CEST NMR Spectra: Towards Studies on a Uniformly C-13-Labeled Protein. *Chembiochem* **14**, 1709-1713 (2013).

19. N. R. Skrynnikov, F. W. Dahlquist, L. E. Kay, Reconstructing NMR spectra of "invisible" excited protein states using HSQC and HMQC experiments. *J Am Chem Soc* **124**, 12352-12360 (2002).

20. R. Auer *et al.*, Measuring the signs of 1H(alpha) chemical shift differences between ground and excited protein states by off-resonance spin-lock R(1rho) NMR spectroscopy. *J Am Chem Soc* **131**, 10832-10833 (2009).

21. A. B. Gopalan, P. Vallurupalli, Measuring the signs of the methyl ^{1}H chemical shift diferences between major and ‘invisible’ minor protein conformational states using methyl ^{1}H multi-quantum spectroscopy. *J Biomol NMR* **70**, 187-202 (2018).

22. T. Yuwen, L. E. Kay, A new class of CEST experiment based on selecting different magnetization components at the start and end of the CEST relaxation element: an application to (1)H CEST. *J Biomol NMR* **70**, 93-102 (2018).

23. T. Yuwen, A. Sekhar, L. E. Kay, Separating dipolar and chemical exchange magnetization transfer processes in ^{1}H-CEST. *Angew Chem Int Ed Engl* **56**, 6122-6125 (2017).

24. P. Vallurupalli, L. E. Kay, Probing slow chemical exchange at carbonyl sites in proteins by chemical exchange saturation transfer NMR spectroscopy. *Angew Chem Int Ed Engl* **52**, 4156-4159 (2013).

25. D. Long, A. Sekhar, L. E. Kay, Triple resonance-based (1)(3)C(alpha) and (1)(3)C(beta) CEST experiments for studies of ms timescale dynamics in proteins. *J Biomol NMR* **60**, 203-208 (2014).

26. A. Kumar, K. Madhurima, A. N. Naganathan, P. Vallurupalli, A. Sekhar, Probing excited state (1)Halpha chemical shifts in intrinsically disordered proteins with a triple resonance-based CEST experiment: Application to a disorder-to-order switch. *Methods* **218**, 198-209 (2023).

27. G. Bouvignies, L. E. Kay, A 2D (1)(3)C-CEST experiment for studying slowly exchanging protein systems using methyl probes: an application to protein folding. *J Biomol NMR* **53**, 303-310 (2012).

28. T. Yuwen, G. Bouvignies, L. E. Kay, Exploring methods to expedite the recording of CEST datasets using selective pulse excitation. *J Magn Reson* **292**, 1-7 (2018).

29. K. H. Gardner, R. Konrat, M. K. Rosen, L. E. Kay, An (H)C(CO)NH-TOCSY pulse scheme for sequential assignment of protonated methyl groups in otherwise deuterated (15)N, (13)C-labeled proteins. *J Biomol NMR* **8**, 351-356 (1996).

30. M. Sattler, J. Schleucher, C. Griesinger, Heteronuclear multidimensional NMR experiments for the structure determination of proteins in solution employing pulsed field gradients. *Progress in Nuclear Magnetic Resonance Spectroscopy* **34**, 93-158 (1999).

31. M. Ikura, L. E. Kay, A. Bax, A novel approach for sequential assignment of 1H, 13C, and 15N spectra of proteins: heteronuclear triple-resonance three-dimensional NMR spectroscopy. Application to calmodulin. *Biochemistry* **29**, 4659-4667 (1990).

32. F. Delaglio *et al.*, NMRPipe - a Multidimensional Spectral Processing System Based on Unix Pipes. *J Biomol NMR* **6**, 277-293 (1995).

33. T. D. Goddard, D. G. Kneller, *SPARKY 3 University of California, San Francisco* (2008).

34. W. Lee, M. Tonelli, J. L. Markley, NMRFAM-SPARKY: enhanced software for biomolecular NMR spectroscopy. *Bioinformatics* **31**, 1325-1327 (2015).

35. A. Ahlner, M. Carlsson, B. H. Jonsson, P. Lundstrom, PINT: a software for integration of peak volumes and extraction of relaxation rates. *J Biomol NMR* **56**, 191-202 (2013).

36. Y. Shen, A. Bax, Protein backbone and sidechain torsion angles predicted from NMR chemical shifts using artificial neural networks. *J Biomol NMR* **56**, 227-241 (2013).

37. M. V. Berjanskii, D. S. Wishart, A simple method to predict protein flexibility using secondary chemical shifts. *J Am Chem Soc* **127**, 14970-14971 (2005).

38. G. Bouvignies, *Chemex (https://github.com/gbouvignies/chemex/releases)* (2012).

39. H. M. McConnell, Reaction Rates by Nuclear Magnetic Resonance. *J Chem Phys* **28**, 430-431 (1958).

40. W. H. Press, B. P. Flannery, S. A. Teukolsky, W. T. Vetterling, *Numerical Recipes in C. The Art of Scientific Computing* (Cambridge University Press, Cambridge (UK), ed. Second Edition, 1992).

41. Y. Shen *et al.*, Consistent blind protein structure generation from NMR chemical shift data. *Proc Natl Acad Sci U S A* **105**, 4685-4690 (2008).

42. A. Cavalli, X. Salvatella, C. M. Dobson, M. Vendruscolo, Protein structure determination from NMR chemical shifts. *Proceedings of the National Academy of Sciences of the United States of America* **104**, 9615-9620 (2007).

43. D. S. Wishart *et al.*, CS23D: a web server for rapid protein structure generation using NMR chemical shifts and sequence data. *Nucleic Acids Research* **36**, W496-W502 (2008).

44. M. Allen, A. Friedler, O. Schon, M. Bycroft, The structure of an FF domain from human HYPA/FBP11. *Journal of Molecular Biology* **323**, 411-416 (2002).

45. M. W. Maciejewski *et al.*, NMRbox: A Resource for Biomolecular NMR Computation. *Biophys J* **112**, 1529-1534 (2017).

46. E. F. Pettersen *et al.*, UCSF Chimera--a visualization system for exploratory research and analysis. *J Comput Chem* **25**, 1605-1612 (2004).

47. R. Bonet, L. Ruiz, E. Aragon, P. Martin-Malpartida, M. J. Macias, NMR structural studies on human p190-A RhoGAPFF1 revealed that domain phosphorylation by the PDGF-receptor alpha requires its previous unfolding. *J Mol Biol* **389**, 230-237 (2009).
